# Single-source formation and assessment of nitrogen-doped graphitic spheres for lithium- and sodium-ion batteries†

**DOI:** 10.1039/d3ra01409f

**Published:** 2023-05-26

**Authors:** Cassius Clark, Christopher A. O'Keefe, Dominic S. Wright, Clare P. Grey

**Affiliations:** a Yusuf Hamied Department of Chemistry Lensfield Road Cambridge CB2 1EW UK cpg27@cam.ac.uk; b Cambridge Graphene Centre 9 JJ Thompson Avenue Cambridge CB3 0FA UK; c The Faraday Institution, Quad One Harwell, Science and Innovation Campus Didcot UK

## Abstract

Optimisation of the annealing time for the fabrication of nitrogen-doped graphitic-spheres (NDGSs), formed from a nitrogen-functionalised aromatic precursor at 800 °C, to give high nitrogen doping has been performed. Thorough analysis of the NDGSs, approximately 3 μm in diameter, pinpointed an optimum annealing time of 6 to 12 hours to obtain highest nitrogen content at the surface of the spheres (reaching a stoichiometry of around C_3_N at the surface and C_9_N in the bulk), with the quantity of sp^2^ and sp^3^ surface nitrogen varying with annealing time. The results suggest that changes in the nitrogen dopant level occur through slow diffusion of the nitrogen throughout the NDGSs, along with reabsorption of nitrogen-based gases produced during annealing. A stable bulk nitrogen dopant level of 9% was revealed in the spheres. The NDGSs performed well as anodes in lithium-ion batteries, providing a capacity of up to 265 mA h g^−1^ at a charging rate of C/20, but did not perform well in sodium-ion batteries without the use of diglyme, consistent with the presence of graphitic regions, but with low internal porosity.

## Abstract 2

The formation of nitrogen-doped graphitic microspheres using a single-source precursor and their use as anodes in lithium- and sodium-ion batteries have been explored.

## Graphical abstract

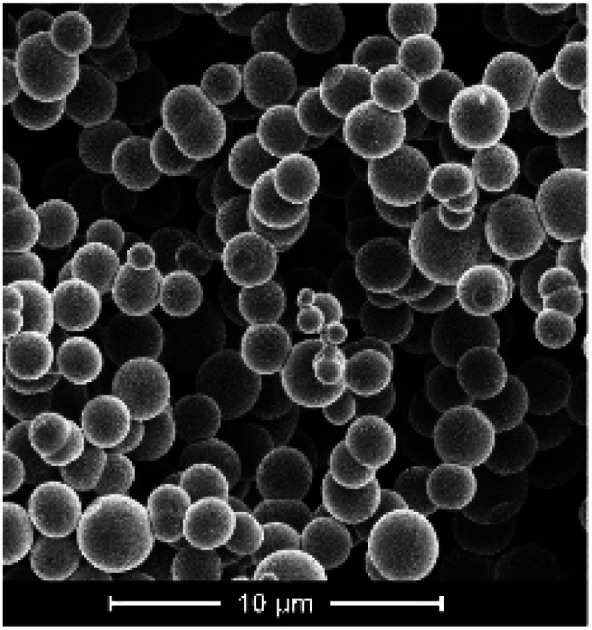

## 1. Introduction

The drive to improve energy density, rate performance and coulombic efficiency in lithium- and sodium-ion batteries has led to the investigation of a wide variety of ordered and disordered carbons. Element-doped graphites, in general, have been studied as next-generation materials for energy storage.^1–3^ Alterations to the graphitic carbon lattice through stoichiometric substitution of carbon atoms with other elements, such as nitrogen, have been shown to be beneficial to electrochemical performance.^1^ For example, reports utilising N-doped graphene as an anode material in lithium-ion batteries (LIBs) demonstrated a capacity of 500 mA h g^−1^ to 900 mA h g^−1^ for a voltage range of 0.01–3 V when cycled at currents of 500 mA g^−1^ and 42 mA g^−1^, respectively.^4–6^ Hard carbons (defined as non-graphitising carbon materials consisting of turbostratic, disordered graphene nanosheets, often described using a ‘house of cards’ model^7–9^), currently represent the standard anode used in sodium-ion batteries (NIBs),^8^ since sodium ions cannot be intercalated into graphite. It has been observed that the use of a diglyme-based electrolyte does, however, allow reversible intercalation of sodium into graphite due to a co-intercalation phenomenon.^10^ In contrast, nitrogen-doped graphite can function as an anode material for sodium-ion batteries.^11–14^ N-doped carbons in the form of both graphene sheets^15^ and hollow graphite spheres^12^ both allow reversible sodium intercalation. This advantageous electrochemical performance is attributed to high internal surface area and porosity and high nitrogen content, specifically N within pyridinic and graphitic environments.^12^ In both LIBs and NIBs, more work is still needed to separate the role of irreversible reactions largely due to electrolyte breakdown at the carbon surfaces *versus* reversible faradaic (insertion) processes on, or in these often quite high surface area materials.

There are a variety of well-known methods for the synthesis of N-doped graphite (NDG), each producing NDG suitable for different purposes.^3^ Pyrolysis is an effective, often single-step, synthetic route. The majority of NDGs produced though pyrolysis involve heating graphene/graphite oxide (GO) with a second, N-containing precursor, such as ammonia, melamine or urea.^2,3,16^ These methods tend to produce flakes, sheets or hollow spheres with high surface area, which, while giving improved capacity when used in a LIB, also result in highly irreversible capacity due to the formation of an extensive solid electrolyte interface (SEI).^5,17^ Graphitic materials with spherical morphology are highly effective for battery applications.^18^ Smooth surfaces and a lack of sharp edges results in improved stability and cycling lifetime, due to avoidance of continuous SEI build-up, which would result in cracking of the anode material and electrolyte consumption.

In this work, a single-step, single-precursor pyrolysis based on synthesis reported previously by us for the production of N-doped sheets of C_3_N,^19^ is explored to reliably produce nitrogen-doped (turbostratic) graphite micro-spheres (NDGSs) for use in energy storage. Our study examines how annealing time affects the morphology and composition of the material, and subsequently how this affects performance as an anode material in LIBs. The NDGSs are characterised using a variety of techniques, analysing the morphology, physical structure, and composition. While our previous study using the same precursor appeared to show the sphere-forming process involved ‘surface bubbling’ and the formation of hollow spheres,^19^ the more in-depth investigation reported here is more consistent with an aerosol process, producing solid spheres. These spheres consist of a turbostratic graphite interior coated with a layer of amorphous carbon. Although the level of nitrogen doping at the surface varies with annealing time (having a maximum composition of *ca.* C_3_N), the nitrogen doping in the bulk of the spheres remains almost constant with a composition of *ca.* C_9_N.

The materials were tested in half-cells *versus* both Li and Na to explore their electrochemical performance. The greater graphitic disorder in samples annealed for shorter times resulted in poorer electrochemical performance in LIBs, whereas the better performance found for extended annealing is probably the result of more complete combustion and rearrangement of the nitrogen within the NDGSs. Despite the high level of nitrogen doping, performance as an anode material in sodium ion batteries (NIBs) was poor, potentially due to a lack of internal porosity, preventing intercalation of sodium, along with the possible formation of an exaggerated SEI, which consumed electrolyte and reduced the capacity.

## 2. Results and discussion

### 2.1 Morphology

In previous work, NDGSs were synthesised by heating the precursor 1,3-phenylenediamine at 800 °C for 7–9 days at an unspecified heating rate,^19^ although a low heating rate of 1 °C min^−1^ is known to promote the preferential formation of sheets.^20^ Here, varying annealing times of 0, 1, 3, 6, 9, 12, and 99 hours (following a ramp over approximately 80 min to 800 °C) were used in order to explore the effects of annealing time on final dopant quantity. In the current work, a higher heating rate of 10 °C min^−1^ was used as it was observed this gave consistent sphere-based morphologically across samples regardless of annealing time and minimised the formation of flakes. The nitrogen-doped graphite (NDG) samples obtained are herein referred to as DG-*X*, where *X* is the annealing time. In the previous study using the same precursor, graphitic flakes were mainly formed, with the NDGSs being a minor component indicating the ramp rate is an important factor influencing morphology.

**Fig. 1:**
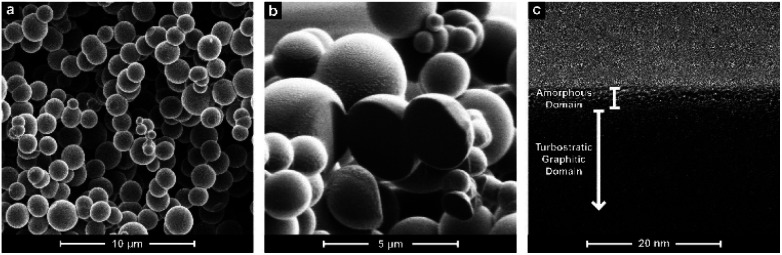
(a) SEM; (b) FIB-SEM; and (c) TEM of DG-12 NDGSs, showing the consistency in shape and filled interior, along with the amorphous and turbostratic graphitic domains within an individual sphere.

Fig. 1a shows the typical morphology of the NDGSs samples (here illustrated with DG-12), consisting of spheres approximately 3 μm in diameter. Some flakes were also observed in the samples (Fig. S1.1.1†). All samples regardless of annealing time produced similar morphologies. Using FIB-SEM, it was observed that the spheres are solid throughout (Fig. 1b), suggesting that they are likely formed through an aerosol process, unlike a bubbling process producing hollow spheres as suggested in the previous study (albeit here using a higher ramp rate).^19^ Individual spheres analysed by TEM (Fig. 1c) show a surface layer of amorphous carbon approximately 5 nm in thickness. Below this layer, turbostratic graphitic domains are apparent, until the spheres are too thick for effective TEM imaging.

The formation of spheres occurs through the initial formation of active carbon chains and subsequent growth of high-molecular-weight residue droplets, which then carbonise.^21^ The concentric arrangement of graphene nano-sheets (Fig. 1c) suggests a liquid–gas interface during the carbonisation process. The surface energy of the droplets is minimised by arranging the basal planes of the sheets to the surroundings.^22^ The outer amorphous carbon layer may form after the carbonisation of the droplets as a secondary process.

### 2.2 Composition

**Fig. 2:**
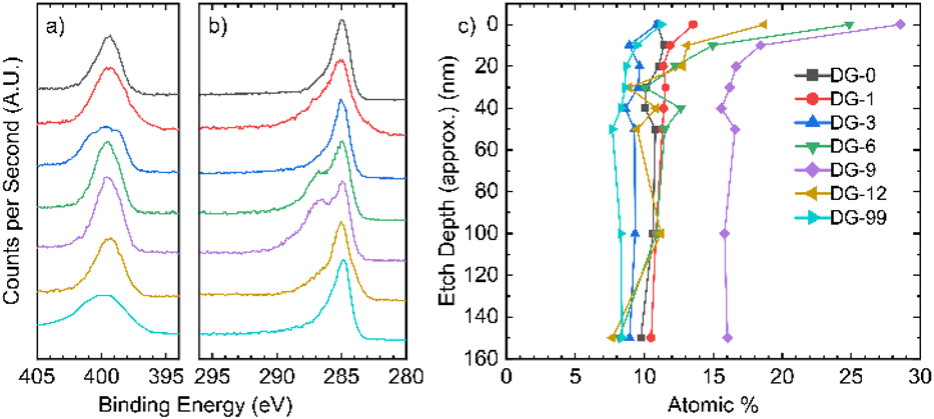
XPS analysis. High resolution spectra of the (a) N 1s region and (b) C 1s region; (c) nitrogen atomic% in all samples as a function of depth.

X-ray photoelectron spectroscopy (XPS) allows analysis of the composition of the elements in the sample, along with the environments they are present in. The XPS survey indicates the presence of carbon, oxygen, and nitrogen for all samples (Fig. S2.1.1†). The N 1s peak broadens and sharpens with changes in the annealing time and follows no discernible trend (Fig. 2a). Deconvolution of the N 1s peaks suggests that a variety of nitrogen environments are present at the surface of all samples, with pyrrolic species being dominant beyond an annealing time of 6 hours (Fig. S2.3.1†). Pyridinic oxides and quaternary (graphitic) nitrogen environments were generally low in concentration. For the C 1s peak (Fig. 2b), a single peak is seen for the samples annealed for short and long times, whilst the samples annealed for intermediate times have a distinct shoulder at higher binding energy.

Depth-profiling XPS was performed on all the NDGS samples annealed for different times. At the surface of the samples, the percentage of nitrogen present was low for both very low and very high annealing times (Fig. 2c). For intermediate times, samples DG-6, 9, and 12, the surface nitrogen content was at its highest. Indeed, for DG-9 the nitrogen content remained high (16%) throughout the sample to the measured depth of approximately 150 nm. Interestingly, in multiple samples annealed for this time period over multiple XPS analyses, the nitrogen content of the 9 hours sample at the surface was at least 28.5%, greater than that of the precursor (25%). This suggests that the nitrogen migrates to the surface of the spheres during annealing, reaching surface saturation after around 9 hours, giving an approximate stoichiometry of C_3_N.

ICP-OES of all samples, however, showed a consistent bulk nitrogen content of *ca.* 9 at%, with hydrogen content of 7.1 at%. Given that the precursor has a 50 at% hydrogen, a high degree of carbonisation is confirmed from this analysis. The fact that there is such a reduction in the nitrogen content for low annealing times shows that even brief carbonisation results in significant loss of nitrogen, which is likely lost as N_2_ or NH_3_.

After this, we propose that nitrogen atoms migrate through the inner bulk towards the surface of the spheres, achieving a stable bulk nitrogen concentration of around 9 at% (approx. C_10_N). Expulsion of nitrogen (as N_2_ and NH_3_) may then occur due to saturation of nitrogen at the surface of the spheres, resulting in the lower nitrogen content for extended annealing times (NG-12 and NG-99). This process gives a roughly uniform nitrogen content at depth for all samples and a gradient in nitrogen content near the surface (<30 nm) for intermediate annealing times (Fig. 2c).

In the high-resolution C 1s spectra (Fig. 2b), a shoulder appears for DG-6 and DG-9, which have the two highest N contents at their surfaces. DG-12 also shows a slight shoulder. In DG-9, this shoulder persists down to a sputtering depth of 150 nm but is not present for DG-6 at any depth below the surface (Fig. S2.2.4†). The high-resolution N 1s spectra (Fig. 2a) shows sharp peaks for these high N content samples; it is suspected that this sharpening is due to the nitrogen saturating on the surface of the carbon spheres as sp^3^ C–N bonded environments. At extended annealing times, the broadening of this sp^3^-peak is due to the release of this nitrogen, resulting in a variety of environments.

**Fig. 3:**
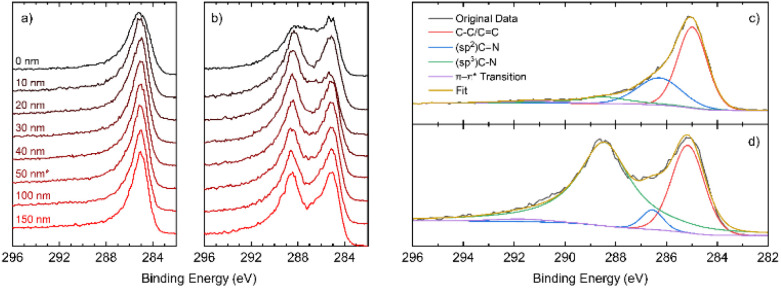
High resolution XPS spectra of the C 1s region for (a) DG-3 and (b) DG-9; high resolution XPS C 1s spectra at a depth of 50 nm with spectral deconvolution for samples annealed for (c) DG-3 (d) DG-9.

In Fig. 3a and b, a comparison of the high-resolution C 1s depth profiles for DG-3 and DG-9 is presented, showing a clear second peak at approximately 289 eV for DG-9. Deconvolution of the spectra shown in Fig. 3c and d allows the assignment of this peak to sp^3^ C–N bonds. Previously, this has been assigned to physisorbed O_2_ on the surface; however, examination of survey spectra (revealing <3% O, compared to 16% N) indicates it is unlikely that oxygen is responsible for this peak.^23^ As the sample is etched to a greater depth, an increase in C–C and C=C environments is observed, coinciding with the observation of stacked nano-sheets and their terminating edges in TEM imagery. However, we see a reduction in the sp^2^ C–N environments, and an increase in sp^3^ C–N environments (Fig. S2.2.12†). This suggests that the nitrogen preferentially takes positions at the surface of graphite nanosheets, as opposed to interstitial dopant positions.

Solid-state nuclear magnetic resonance spectroscopy gives further insight into the carbonisation of the material. A general trend of proton removal was observed (Fig. S.3.1.2†) with increasing annealing time, the ^13^C NMR spectra showing a resonance at a chemical shift consistent with a disordered graphitic carbon. This trend is as expected but reinforces the conclusion of continued carbonisation, that is, decomposition of C–H bonds to form C–C bonds with extended time at high temperature.

### 2.3 Structure

**Fig. 4:**
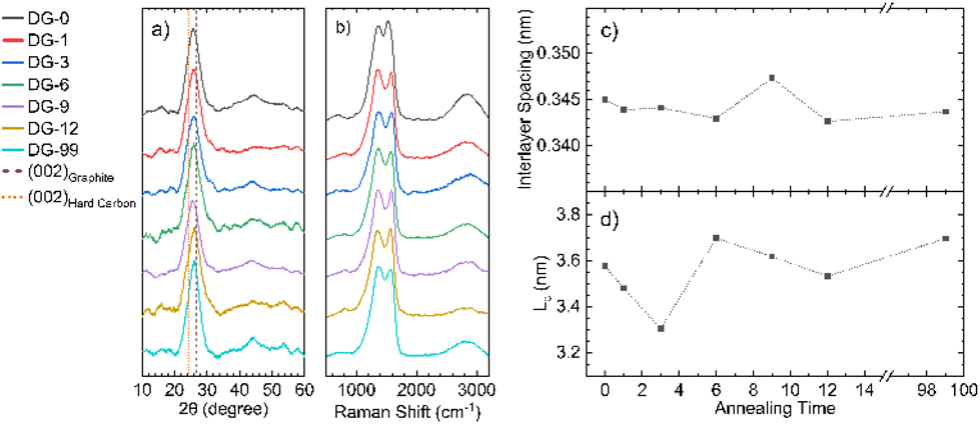
X-ray diffraction and Raman spectroscopic analysis. (a) Diffraction patterns for NDG samples annealed for different times showing the (002) peak and very broad (010) region, lines indicating the expected positions for the (002) peak for graphite and typical hard carbons are included;^9^ (b) Raman spectra of NDG samples, showing typical D and G peaks, along with a broad second-order peak region from 2300 to 3200 cm^−1^; graphs of (c) interlayer spacing and (d) interlayer crystallite size (*L*_c_), calculated using the Bragg and Scherrer equations (Scherrer constant, *K* = 0.89 (ref. 33)), respectively.

Powder X-ray diffraction (pXRD) for all samples annealed at different temperatures shows a typical pattern for carbons formed through pyrolysis (Fig. 4a), with a strong (002) peak at approximately 24° 2*θ* and a broad 10 peak (consisting of the (100) and (010) peaks) at around 45° 2*θ*. Use of the Bragg and Scherrer equations allowed estimations of interlayer spacing and crystallite size (Fig. 4c and d). The graphitic layers have already formed during the ramping process and no major change in interlayer spacing with annealing time was observed; however, the average spacing was 0.01 nm greater than that seen for crystalline graphite (0.335 nm), likely due to a combination of disordered layers and interstitial doping. This *d*-spacing is also significantly smaller than that of hard carbons (>0.367 nm), which are able to reversibly intercalate Na^+^. The average crystallite size in the direction normal to the graphite sheets (*L*_c_) also shows no definite trend. However, NDGs formed using a shorter annealing time gave generally smaller sizes. Meaningful calculation of the crystallite size along the *a*-axis was not possible due to low signal-to-noise ratios of the data, suggesting a small value of the in-plane crystalline size (*L*_a_).

Raman spectroscopy was used to evaluate the degree of structural disorder of the samples (Fig. 4b). The D peak (∼1350 cm^−1^) and the G peak (∼1580 cm^−1^), typical of disordered graphitic structures are observed, along with a broad peak above 2300 cm^−1^, consisting of the second-order peaks, primarily the 2D peak (Fig. 4b). A high (>1) value of the intensity ratio of the D and G peaks (*I*(D)/*I*(G)) indicates a high level of disorder within the NDG samples, as the D peak is only produced near to defects, including edges. Use of the relation set out by Ferrari *et al.* allows for a calculation of *L*_a_:^24^
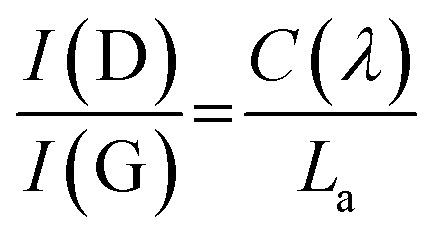


*L*_a_ values showed no discernible trend (Fig. S4.1†), an average *L*_a_ of 15.4 ± 0.7 nm was calculated.

With increasing annealing time, the full-width half-maxima (FWHM) of the G peak, FWHM(G), increases for up to sample DG-6, after which is remains approximately the same (Fig. S4.2a†). This trend is attributed to a process of defect distribution occurring during the initial hours of annealing, producing variations in local C–C and C–N environments, resulting in a broader distribution of G-peak energies. A general consistency of the FWHM(D) is observed indicating the overall defect (including dopant) quantity remains roughly the same (Fig. S4.2b†), consistent with ICP-OES. The merging of the G peak with the defect-activated D′ mode at ≈1620 cm^−1^ results in an apparent shift of the G peak position (Pos(G)) to higher frequencies compared to pristine graphite (1580 cm^−1^).^25^ Increased annealing time gives a decrease in Pos(G) due to increased carbonisation (Fig. S4.2c†), although not down to 1580 cm^−1^ as defects are still present.

### 2.4 Electrochemistry

**Fig. 5:**
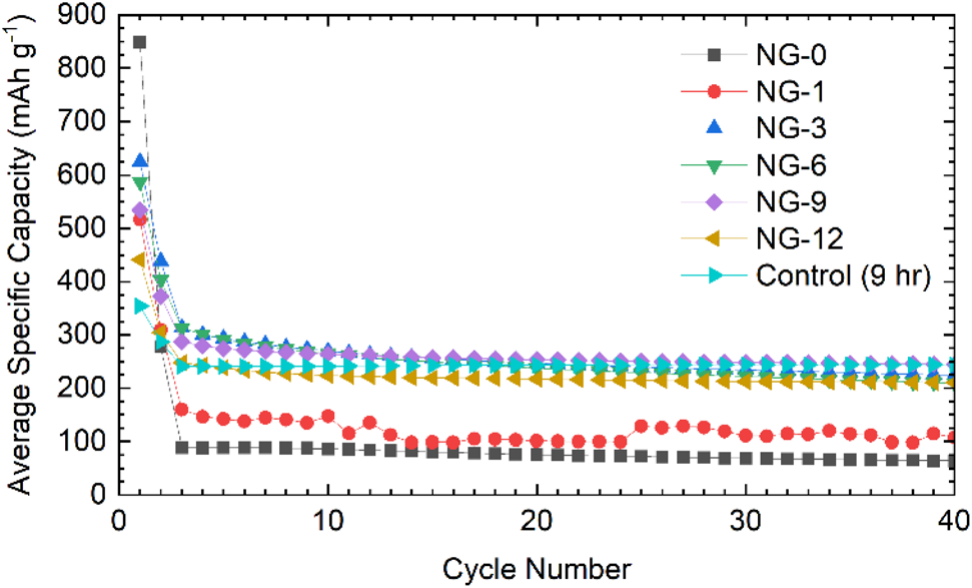
The electrochemical performance of NDGS materials annealed from 0–12 hours in lithium half-cells. Material annealed for short amounts of time performed significantly worse than those annealed for longer times.

**Fig. 6:**
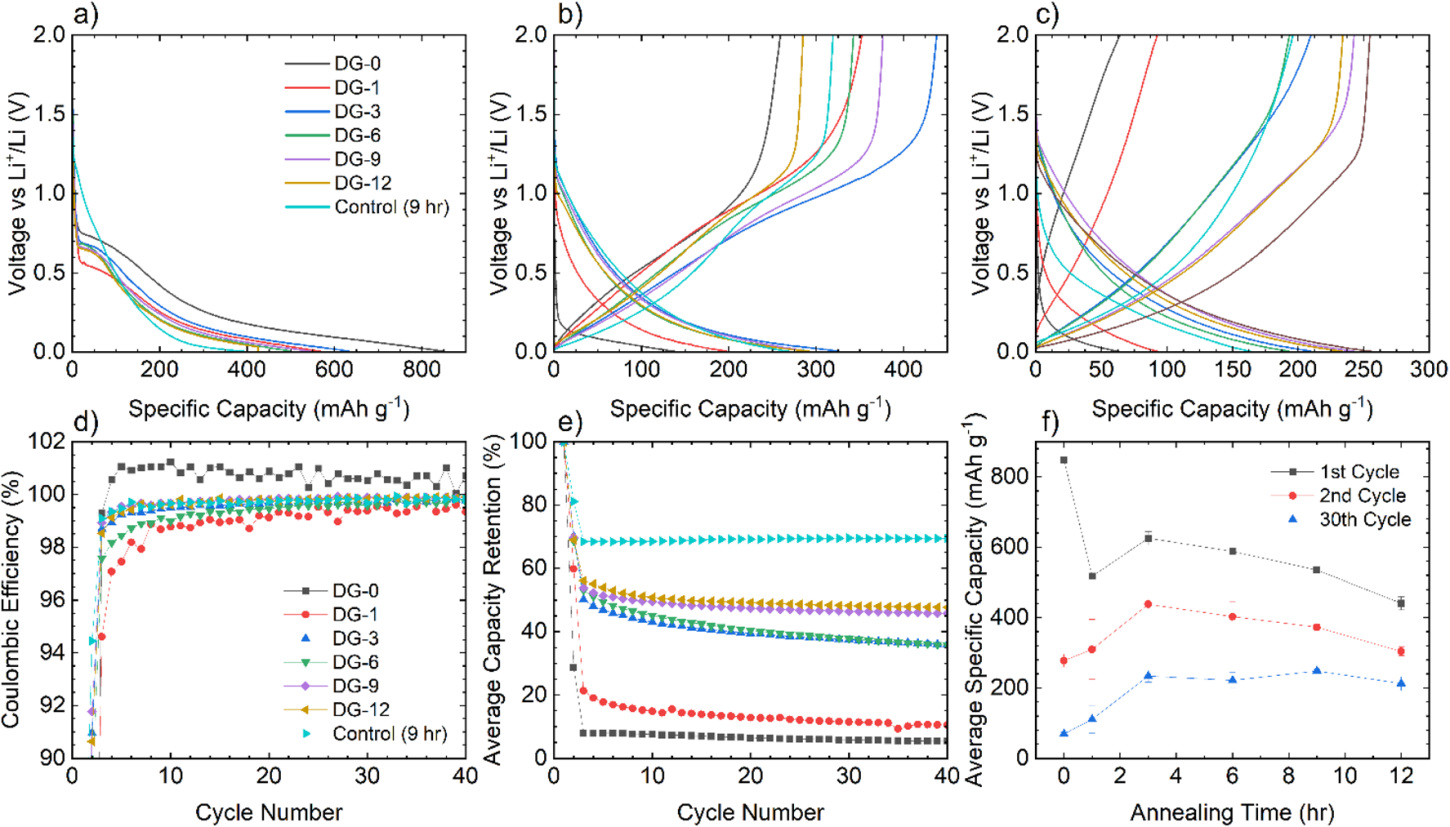
Electrochemical data for NDGS materials cycled in lithium-ion half-cells using 1 M LiPF_6_ in EC/DMC (50/50 v/v) as the electrolyte. Exemplar voltage profiles for all materials for the (a) first, (b) second, and (c) thirtieth cycles are plotted. (d) The average coulombic efficiency for the NDGS cells. High efficiencies were achieved at extended cycling for all cells, but major capacity losses occurred in the initial cycles. (e) The average capacity retention for the NDGS cells, longer annealing times leading to generally greater capacity retention. (f) The average specific capacity across annealing time for selected cycles. Intermediate-extended annealing times generally had higher overall performance.

The NDGSs were cast in to electrodes with 10 wt% Carbon Black and polyvinylidene fluoride (PVDF) as a binder. They were cycled in lithium- and sodium-ion half-cells with the performance of samples, DG-0–DG-12, in lithium-ion half-cells shown in Fig. 5. Cells made from samples that were annealed for a short amount of time had significantly worse capacity retention and this was observable across multiple cells (Fig. 6e and f). For all cells, the greater capacity seen in the first discharge can be attributed to the formation of a solid electrolyte interphase, along with a slower first cycle charging rate.

The lithiation mechanism in hard carbons is separated into two distinct regions: a ‘sloping region’, at voltages above 0.1 V, in which lithium ions occupy defect sites and edges of the disordered graphitic sheets, and a ‘plateau region’, at voltages near 0 V, where Li ions form small Li clusters or “pools” within the internal nanopores. Intercalation of Li between the disordered graphene-like carbon sheets occurs in both the sloping and plateau regions, but in the former, the associated electrons are associated with defects on the carbon sheets. For lithiation of hard carbons, the plateau region is more pronounced at slow charging rates.^9,26–28^ In ordered graphite, the intercalation mechanism of lithiation has distinct stages at specific voltages, corresponding to phase transformations. These stages all occur below 0.2 V.^29^

The voltage profiles for cells for cycles 1, 2, and 30 can be seen in Fig. 6a–c. For the first cycle, no capacity is achieved until 0.7 V, at which point a plateau is observed attributed to the formation of an SEI,^30^ followed by a slope down to full lithiation at 5 mV with no discernible features. These processes can be observed clearly in the d*Q*/d*V* plots (shown in Fig. S5.1†).

After the first cycle, a featureless slope is seen in the voltage profiles for all samples regardless of annealing time (again apparent in the d*Q*/d*V* plots (Fig. S5.1†)). This indicates that lithiation is not occurring in distinct stages, but as a continuous, undefined process. This is due to the disorder in the materials and suggests that they are performing in a similar manner to hard carbons. In this case, the majority of the capacity is coming from lithium adsorption on and between the disordered carbon sheets containing multiple defect sites, including topological defects from nitrogen atoms. The disordered nature of sheets within the NDGSs (as is visible in TEM images) produces the observed sloping profile due to electrochemically inequivalent sites.^27^ The lack of a low voltage plateau, expected at around 100 mV, is consistent with the lack of internal porosity (as seen for these materials *via* TEM).

Samples with an annealing time above 3 hours show reasonable reversibility, with a coulombic efficiency of >99.5% after the 10th cycle (Fig. 6d). Samples DG-0 and DG-1 have significantly lower capacity after the first cycle compared to other samples, with first cycle coulombic efficiencies of 33% and 63%, respectively. This is significantly lower than the other DG samples, with had first cycle coulombic efficiencies of ∼70%. This may be due to the lower level of carbonisation and higher level of disorder of the short-annealing time samples, observed from a higher FWHM of the G band by Raman spectroscopy (Fig. S4.2a†), and from the normalised ^1^H integral from the solid-state NMR (Fig. S3.1.2†), giving a lower conductivity, affecting their ability to perform as anode materials. Poor lithium diffusion kinetics are suggested by the steep voltage profile during charge. This may be due to low porosity and formation of a thick SEI with high impedance, which would contribute to capacity loss.^4,31^ The undoped control sample had the greatest first cycle coulombic efficiency, suggesting that nitrogen dopants may cause additional electrolyte decomposition. However, DG-0 settles to a CE of greater than 100% after 5 cycles, suggesting some degradation of SEI is occurring during the charge (delithiation) process. When CMC was used as a binder, sample DG-1 showed the greatest rate performance (Fig. S5.3†), whilst samples DG-0, DG-3 and DG-6 all had worse performance. DG-1 and DG-6 had similar, sloping voltage profiles, whilst DG-0 and DG-3 had steeper profiles. This observation suggests that an interaction between the binder and DG samples occurs, that changes the kinetics. While the results for DG1 are encouraging, the variation in electrochemical results between samples clearly shows that further optimisation of the binder, the slurry making processes and the additives in the electrolytes is clearly need to understand the surface–electrolyte interactions and reactivity and to ultimately improve battery performance.

**Fig. 7:**
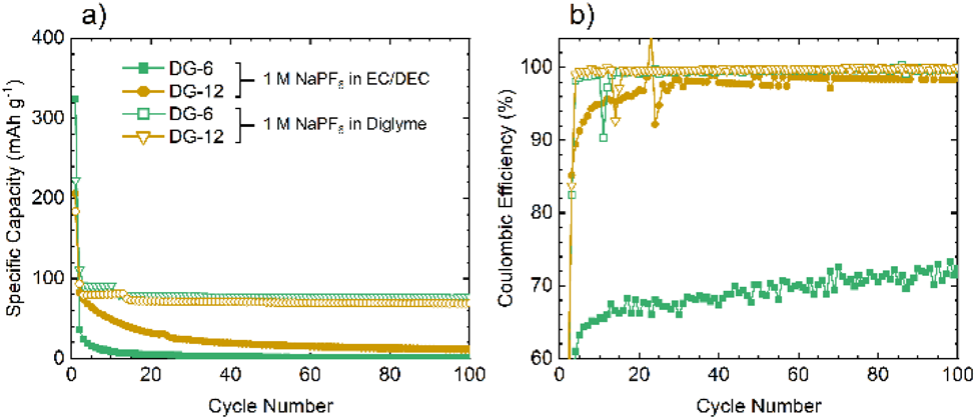
Comparison of the performances of DG-6 and DG-12 when used in Na half-cells using an electrolyte of 1 M NaPF_6_ in EC/DEC (50/50 v/v) electrolyte or 1 M solution of NaPF_6_ in diglyme. (a) The specific capacity showing greater intercalation ability when using diglyme as the electrolyte solvent. (b) The coulombic efficiency demonstrating greater reversibility when utilising diglyme as the electrolyte solvent. All cells were run at C/20 within a potential range of 0.005–2 V *vs.* Na^+^/Na.

Given the similarities between the materials discussed here and hard carbons, select materials, DG-6 and DG-12, were tested in Na half-cells (Fig. 7a). The cells fabricated using NaPF_6_ and EC/DEC as the electrolyte gave very poor performance, with a >90% capacity loss by cycle 3 (C/20). Moderate capacity was observed for the first discharge (sodiation) with a capacity of 359 mA h g^−1^ for DG-6 and 206 mA h g^−1^ for DG-12. However, subsequent charging and further cycles had a significantly reduced capacity (a minimum 64% capacity loss) indicating consumption of electrolyte and the formation of an SEI. The poor capacity was tentatively ascribed to the noticeably smaller interlayer spacings between the carbon sheets (Fig. 4c) compared to those determined by Stratford *et al.*^9^ for a series of hard carbons, preventing Na^+^-ion intercalation. Previous studies have utilised diglyme as a co-intercalation agent,^8^ allowing Na^+^-ions to be co-intercalated between graphite sheets. This strategy was used here in attempts to increase capacity. Altering the electrolyte solvent to diglyme improved the performance of all samples, without any particular trend (Fig. 7b). It can be noted that the minor ‘step’ features below cycle 25 in the specific capacity data using glyme (Fig. 7a) are an artifact due to unexpected but major ambient temperature fluctuation during cycling. After this, a return to stable, room-temperature conditions gave cycling with consistent, continuous capacities.

Improvements seen using diglyme as the solvent indicate that the poor performance without diglyme is due to the presence of (non-porous) graphitic layers. The diglyme can act as a co-intercalation agent, enabling effective reversible intercalation of Na^+^ into and out of the graphitic layers. Capacities observed here, between 50–80 mA h g^−1^, are slightly lower, but consistent with those seen in the literature for graphite/Na^+^/diglyme systems (∼90 mA h g^−1^).^10^ The results suggest that these disordered carbons comprise of graphitic regions that are sufficiently ordered to allow sufficient expansion of the interlayer spacing to allow co-intercalation of the glyme-coordinated Na^+^ ions.

## 3. Conclusions

Nitrogen-doped solid graphitic spheres were successfully synthesised using a commercially available nitrogen-functionalised aromatic precursor. Optimisation of the annealing duration identified that an annealing time of between 6–12 hours gave the highest nitrogen doping at the surface with a stoichiometry of approximately C_3_N. However, thorough characterisation, including depth-profiling XPS and ICP-OES, showed that in the bulk, little difference is seen in nitrogen content across annealing times, with results indicating that C_9_N is the stable quantity of doped-nitrogen in the bulk achieved using this method. XPS further revealed variation in the quantity of sp^3^ and sp^2^ C–N environments present, dependant on both annealing time and material depth. Intermediate annealing times of 6–12 hours showed higher sp^3^ C–N presence at the surface. The 9 hour sample was the only one that showed major sp^3^ C–N character in the bulk.

It is suggested that variation in nitrogen quantity across annealing times occurs through slow dissociation of nitrogen from the centre of the spheres outwards, giving a high surface nitrogen content at intermediate annealing times. Subsequent surface saturation and loss of nitrogen results in reduced nitrogen quantity for high annealing times at the surface.

The NDGS materials were tested in both lithium- and sodium-ion batteries. In lithium half-cells, the materials gave modest capacities close to that of graphite with adequate capacity retentions. The voltage profiles were sloping without clear plateaus, indicating that lithiation occurs not *via* a staged mechanism, but instead, due to the materials disorder, as a single, continuous, ill-defined process. This is consisted with the disordered layered structure visible in TEM images. The low voltage process often seen in hard-carbons was not observed, consistent with an observed low internal porosity. The materials performed poorly in sodium half-cells, with high-capacity losses during the initial cycles. However, use of the co-intercalation agent diglyme gave moderate improvement, suggesting that the N-doped graphitic sheets can be intercalated with the use of an appropriate cosolvent. These results suggest the NDGS materials comprise disordered graphitic sheets, allowing lithium-ion intercalation but being unfavourable to sodium-ion intercalation in the absence of solvent co-intercalation.

## 4. Experimental details

### 4.1 Synthesis of NDGS

1,3-Phenylenediamine (200 mg) (Sigma-Aldrich) was inserted into a quartz ampoule (*L*: 100 mm *Ø*: 25 mm) under inert atmosphere (N_2_, O_2_ < 0.5 ppm, H_2_O < 0.5 ppm). The ampoule was sealed with hydrogen/oxygen torch under dynamic vacuum (1 × 10^−3^ Bar). The ampoules were heated inside of a Carbolite ELF 11/14B furnace at 10 °C min^−1^ to 800 °C and held at this temperature for specified amounts of time, ranging from 0 hours (with immediate cooling) to 99 hours. Material was extracted by scoring with a diamond glass cutter and snapping the ampoules. The ampoules were opened in air using a custom ‘leverage-snapping’ tool, due to the high-pressure nature of the annealed ampoules, allowing the ampoules to be opened at a safe distance (see ESI S5.1†). The carbonaceous material produced was then washed with water and acetone to remove by-products, and then allowed to dry for 12 hours under ambient conditions. Typically, approximately 80 mg of material was produced.

### 4.2 Analytical techniques

Scanning electron microscope (SEM) images were collected using a Magellan 400 at 2 kV. Transmission electron microscope (TEM) images were collected using a Thermo Scientific (FEI) Talos F200X G2 TEM.

Powder X-ray diffraction data were collected on a Malvern Panalytical X'Pert Pro, using an X'celerator detector with non-monochromated CuKα radiation (*λ* = 1.5418 Å) The sample was placed on a glass holder and measured in reflection geometry with sample spinning. A 2*θ* range of 5–90° was used, with an effective step size of 0.167°. The collection time was 20 minutes. Raman spectroscopy mapping measurements were performed using a Renishaw InVia with a 2400 l mm^−1^ grating and 514 nm laser. Peak fitting was performed using OriginLab Origin data analysis software using a Gaussian fit. A predetermined background was removed, and the pattern was smoothed using a Savitzky–Golay method using a window of 50 points and a polynomial order of 1.

XPS data was acquired using a Kratos Axis SUPRA using monochromated Al kα (1486.69 eV) X-rays at 15 mA emission and 12 kV HT (180 W) and a spot size/analysis area of 700 × 300 μm. The instrument was calibrated to gold metal Au 4f (83.95 eV) and dispersion adjusted to give a BE of 932.6 eV for the Cu 2p_3/2_ line of metallic copper. The Ag 3d_5/2_ line full-width half-maxima (FWHM) at 10 eV pass energy was 0.544 eV. Source resolution for monochromatic Al Kα X-rays was ≈0.3 eV. The instrumental resolution was determined to be 0.29 eV at 10 eV pass energy using the Fermi edge of the valence band for metallic silver. Resolution with charge compensation system on <1.33 eV FWHM on PTFE. High-resolution spectra were obtained using a pass energy of 20 eV, step size of 0.1 eV and sweep time of 60s, resulting in a line width of 0.696 eV for Au 4f_7/2_. Survey spectra were obtained using a pass energy of 160 eV. Charge neutralisation was achieved using an electron flood gun with filament current = 0.4 A, charge balance = 2 V, filament bias = 4.2 V. Successful neutralisation was judged by analysing the C 1s region wherein a sharp peak with no lower BE structure was obtained. Spectra have been charge-corrected to the main line of the carbon 1s spectrum (adventitious carbon) set to 284.8 eV. All data were recorded at a base pressure of below 9 × 10^−9^ Torr and room temperature of 294 K. Data were analysed using CasaXPS v2.3.19PR1.0. The spectra were calibrated for the carbon C 1s excitation at a binding energy of 285 eV. Peaks were fitted with a Shirley background prior to component analysis. Gas cluster-ion etching was performed using an Ar_1000_^+^ ion cluster accelerated through 10 keV over a 2 mm^2^ raster area.

For elemental analysis using inductively couple plasma optical emission spectroscopy (ICP-OES), samples were suspended in sulfuric acid (3.75 mL) and 30% H_2_O_2_ solution (1.25 mL), added dropwise. Samples were dissolved by heating at 80 °C for 30 minutes, then diluted with water (5 mL). A 0.5 mL aliquot of each was diluted to 10 mL with water.Samples were run and analysed on a Thermo Fisher Scientific iCAP7400 Duo ICP-OES spectrometer using Qtegra software, calibrated against a standard curve constructed from a series of dilutions of a commercial ICP standard with a 2% nitric acid solution. ICP standards and 30% hydrogen peroxide were purchased from Sigma-Aldrich. Nitric acid and sulfuric acid (Trace Metal grade) were purchased from Fisher.

Solid-state NMR (SSNMR) was performed on a Bruker Avance IIIHD spectrometer equipped with a 11.7 T magnet (*ν*_0_(^1^H) = 500 MHz, *ν*_0_(^13^C) = 125.8 MHz). A 1.3 mm double-channel MAS probe was used with spinning speed of 55 kHz using a Hahn-echo pulse sequence. ^1^H spin-lattice relaxation time constants (*T*_1_) were measured using a saturation recovery experiment and recycle delays were set to five times the longest *T*_1_ to ensure quantitative ^1^H spectra.

### 4.3 Cell fabrication and electrochemical cycling

NDGSs (80 wt%) were mixed with Super P Carbon Black (Timcal) (10 wt%) and polyvinylidene fluoride (PVDF) (10 wt%) in *N*-methyl-2pyrrolidone (NMP) (Sigma-Aldrich), homogenised in an Intertronics ThinkyMixer at 2000 rpm for 10 minutes. The resulting slurry was spread over copper foil at a thickness of 300 μm, dried under vacuum at 100 °C, and cut into electrodes (*Ø*: 12.5 mm). For electrochemical cycling, the electrodes were subsequently tested in CR2032 coin cell for lithium- or sodium-ion half-cell configurations. The electrolyte was a 1 M solution of either LiPF_6_ (Sigma-Aldrich) in a 1 : 1 volume ratio of ethylene carbonate (EC) : dimethyl carbonate (DMC) or NaPF_6_ (made in-house, reported in ref. 32), in a 1 : 1 volume ratio of ethylene carbonate (EC) : diethyl carbonate (DEC), for lithium or sodium cells respectively. Alternatively, sodium half-cells utilising diglyme as a co-intercalation agent used a 1 M solution of NaPF_6_ in diglyme as the electrolyte. The counter electrode was either pure lithium or sodium metal, as appropriate. The separator was Whatman 1 glass fibre. Once assembled, the cells were rested for 24 hours before cycling at C/100 for the first cycle then C/20 for further cycles, calculated using a theoretical specific capacity of graphite (372 mA h g^−1^ for lithium cells, and 300 mA h g^−1^ as an estimation for sodium cells). Fast-charging measurements cycled at higher rates are as specified in the main text.

## Author contributions

Cassius Clark: conceptualisation, methodology, software, validation, formal analysis, investigation, data curation, writing – original draft, visualisation. Christopher A O'Keefe: formal analysis, writing – reviewing and editing, supervision. Dominic S Wright: conceptualisation, writing – reviewing and editing, supervision. Clare P Grey: conceptualisation, writing – reviewing and editing, supervision.

## Conflicts of interest

There are no conflicts of interest to declare.

## Supplementary Material

RA-013-D3RA01409F-s001
