# Supplementary material for: Single-source formation and assessment of nitrogen-doped graphitic spheres for lithium- and sodium-ion batteries
Source: RSC Adv. 2023 May 26;13(23):15918–25. doi: 10.1039/d3ra01409f (PMC10214001; doi:10.1039/d3ra01409f)
Supplement: RA-013-D3RA01409F-s001 [file RA-013-D3RA01409F-s001.pdf]

Electronic Supplementary Material (ESI) for RSC Advances.
This journal is © The Royal Society of Chemistry 2023

Supplementary Information:

Formation and characterisation of single-source nitrogen-doped graphitic spheres for use in lithium-ion batteries

Cassius Clark [a,b,*], *Christopher A. O'Keefe* [a], *Dominic S. Wright* [a,c] *and Clare P. Grey* [a,b,c]

[a] Yusuf Hamied Department of Chemistry, University of Cambridge, Lensfield Road, Cambridge, CB2 1EW, UK

[b] Cambridge Graphene Centre, University of Cambridge, 9 JJ Thomson Avenue, Cambridge, CB3 0FA, UK

[c] The Faraday Institution, Quad One, Harwell, Science and Innovation Campus, Didcot, UK

Email: cc986@cam.ac.uk, co402@cam.ac.uk, dsw1000@cam.ac.uk, cpg27@cam.ac.uk

S1. Microscopy

S1.1 Scanning Electron Microscopy

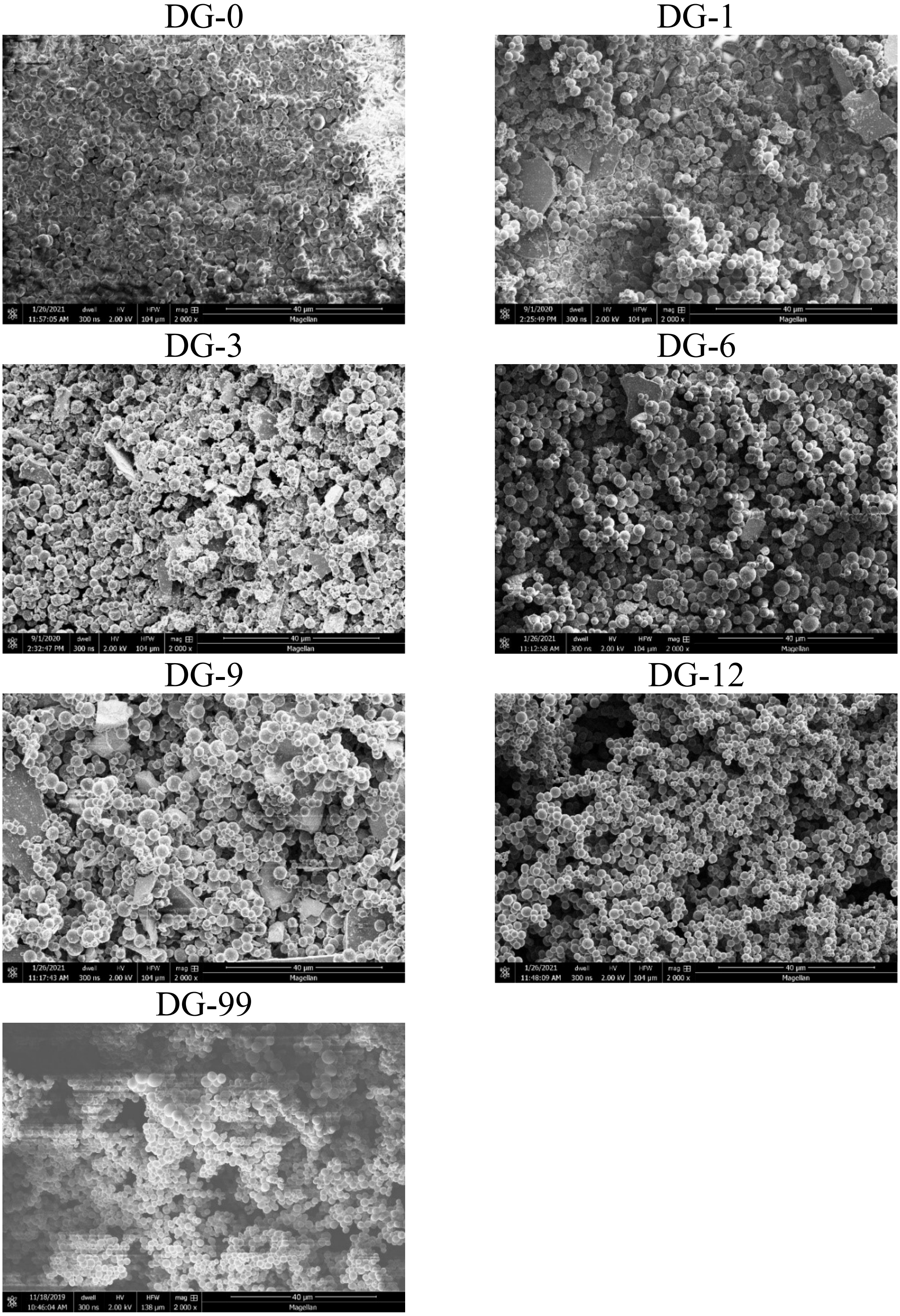

Fig. S1.1.1 SEM images of all of the samples taken at 2000x magnification, showing how in all cases the material is composed of almost entirely carbon spheres.

S1.2 Transmission Electron Microscopy

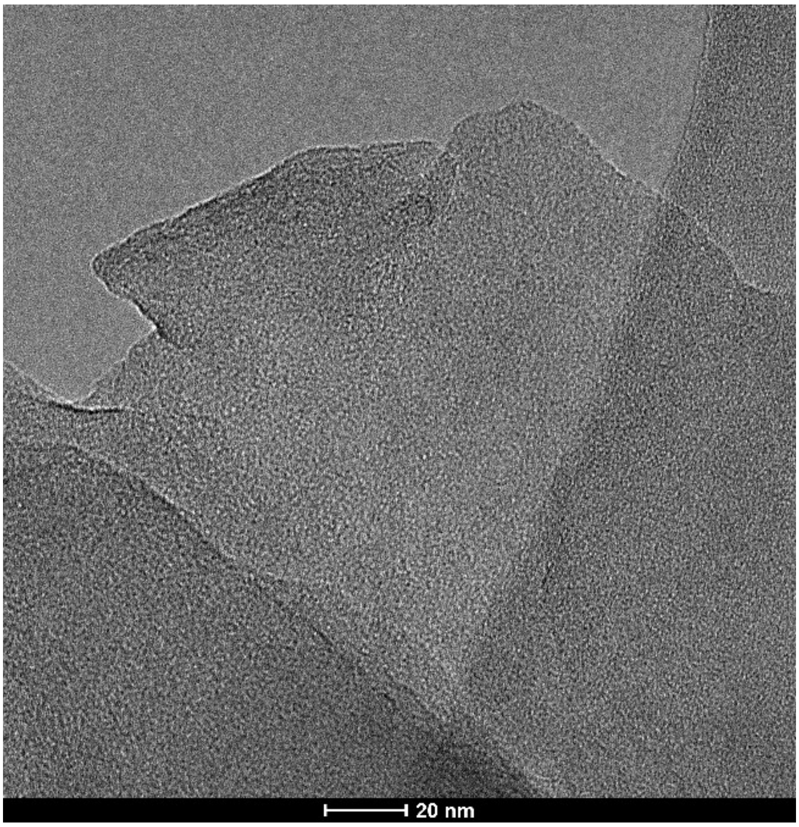

Fig. S1.2.1 TEM image showing the amorphous nature of flakes present amongst the spheres in the material.

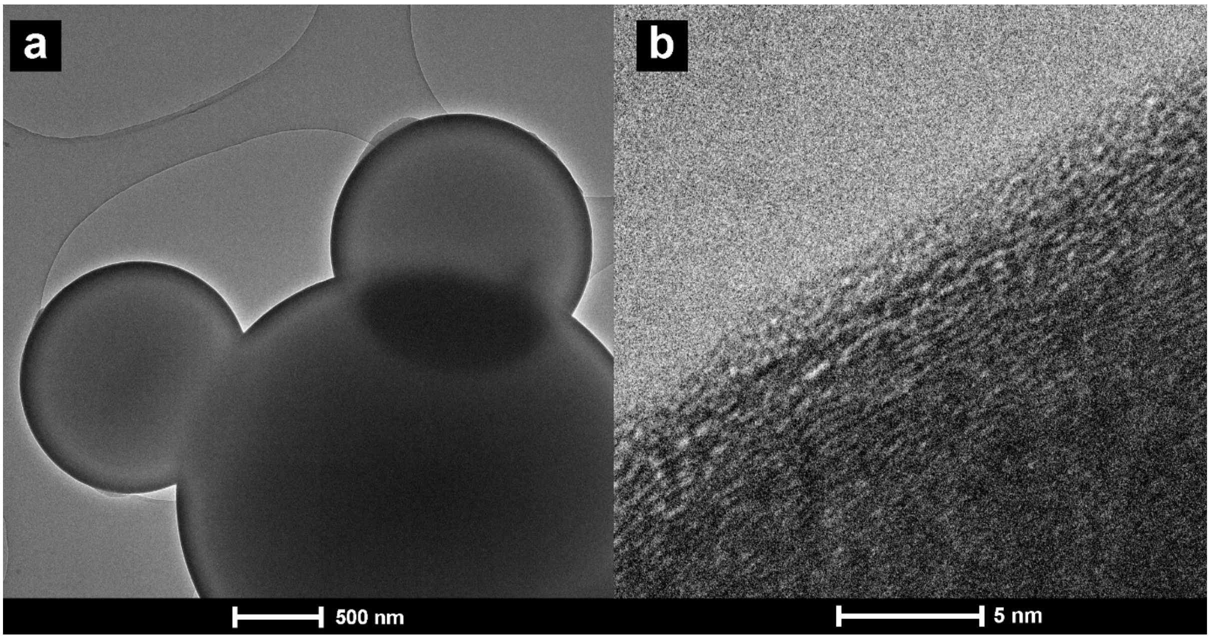

Fig. S1.2.2 TEM images of a) Conjoined spheres, the TEM grid can be seen in the background. b) the surface of the spheres, demonstrating the disordered layers present.

S2. X-ray Photoelectron Spectroscopy

S2.1 Full Survey Scan

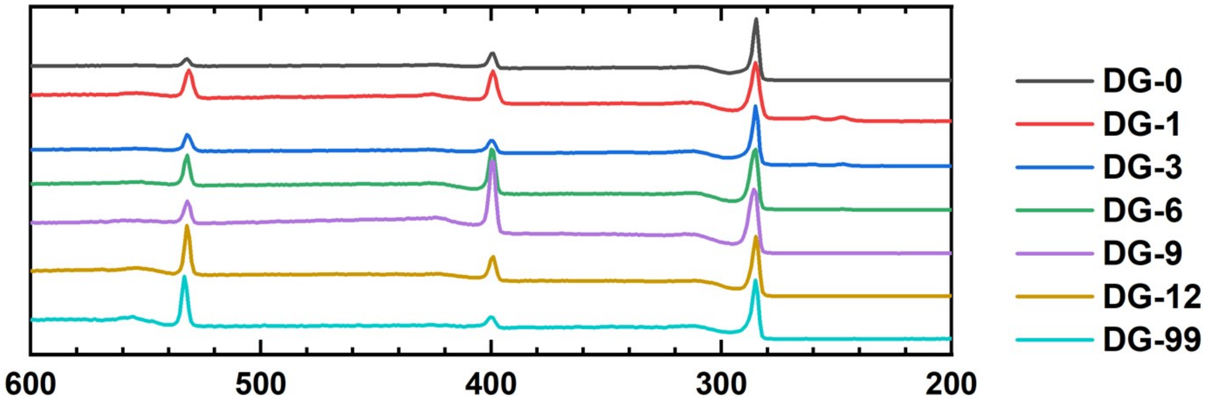

Fig. S2.1.1 Overall survey scans for all samples allowing the quantification of elements at the surface. The O1s (983 eV), N1s (399 eV) and C1s (284 eV) peaks can be seen.

S2.2 High Resolution C1s Sputtered Depth Scans

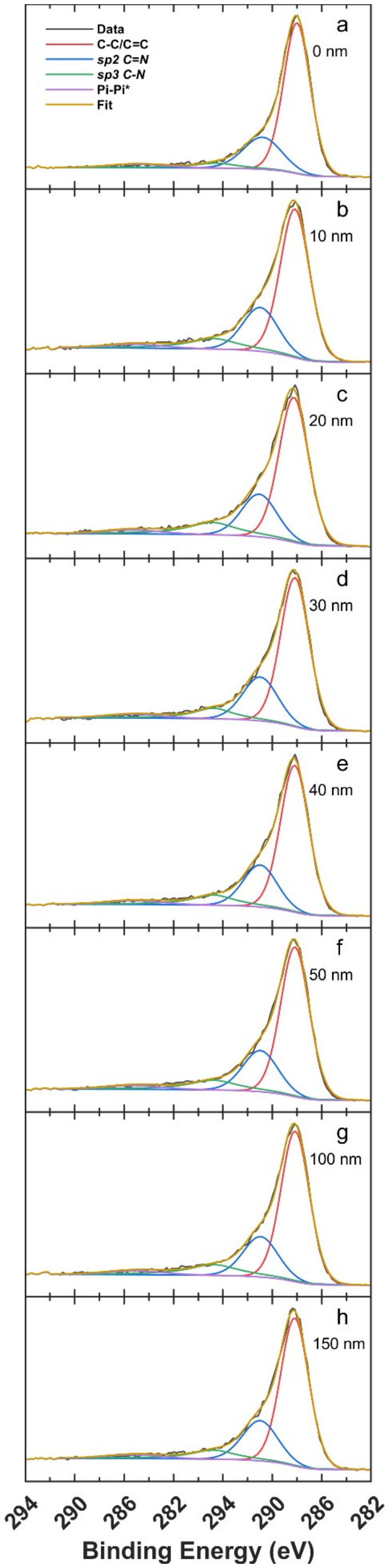

Fig. S2.2.1. For sample DG-0: deconvoluted high resolution C1s scans at depths of a) surface; b) 10 nm; c) 20 nm; d) 30 nm; e) 40 nm; f) 50 nm; g) 100 nm; h) 150 nm.

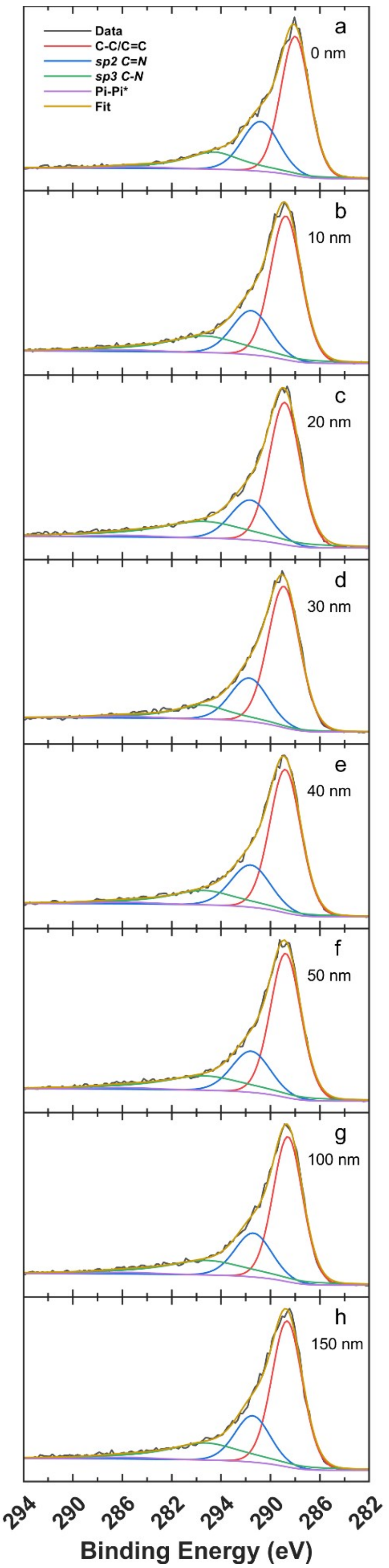

Fig. S2.2.2. For sample DG-1: deconvoluted high resolution C1s scans at depths of a) surface; b) 10 nm; c) 20 nm; d) 30 nm; e) 40 nm; f) 50 nm; g) 100 nm; h) 150 nm.

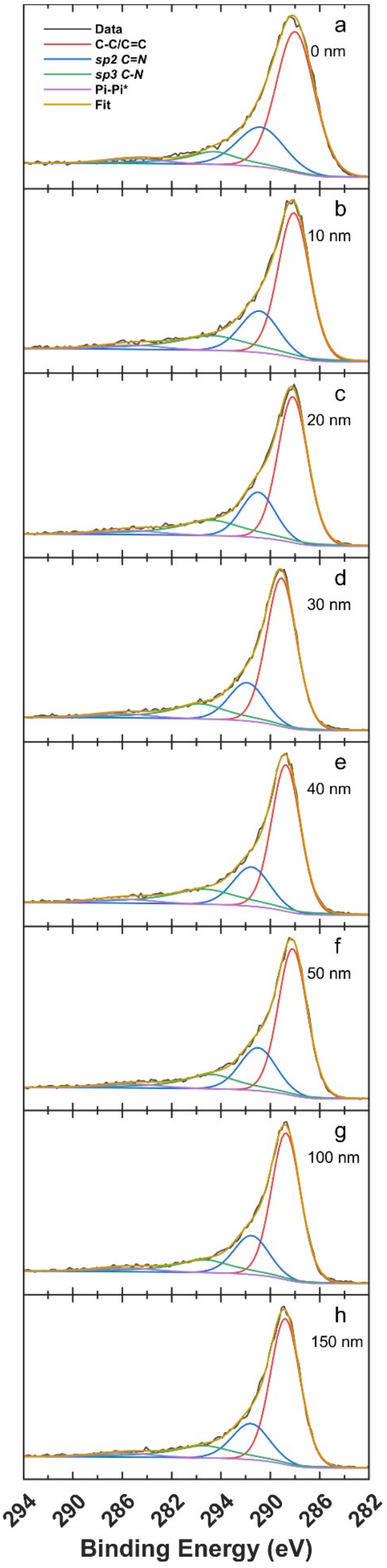

Fig. S2.2.3. For sample DG-3: deconvoluted high resolution C1s scans at depths of a) surface; b) 10 nm; c) 20 nm; d) 30 nm; e) 40 nm; f) 50 nm; g) 100 nm; h) 150 nm.

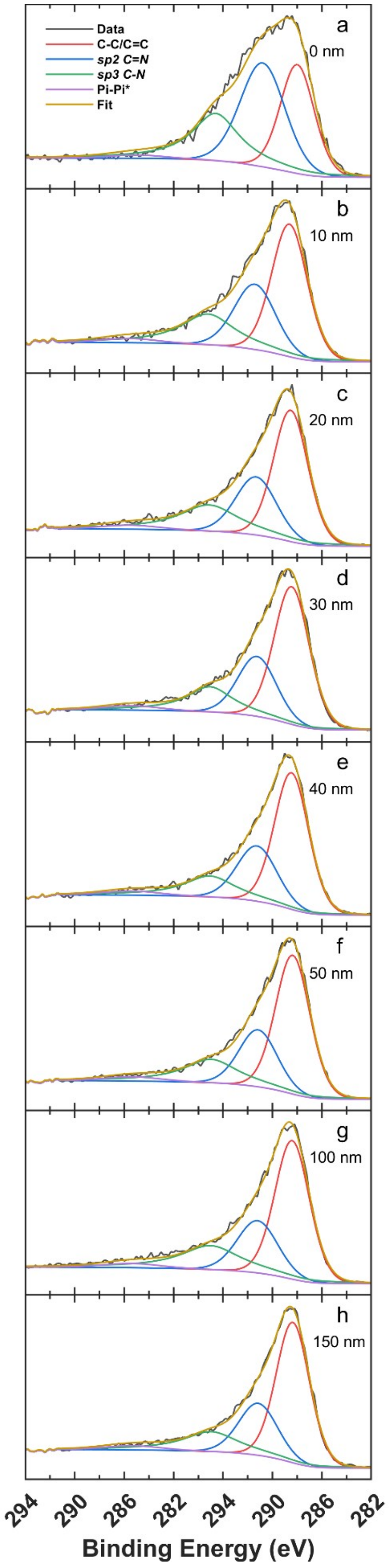

Fig. S2.2.4. For sample DG-6: deconvoluted high resolution C1s scans at depths of a) surface; b) 10 nm; c) 20 nm; d) 30 nm; e) 40 nm; f) 50 nm; g) 100 nm; h) 150 nm.

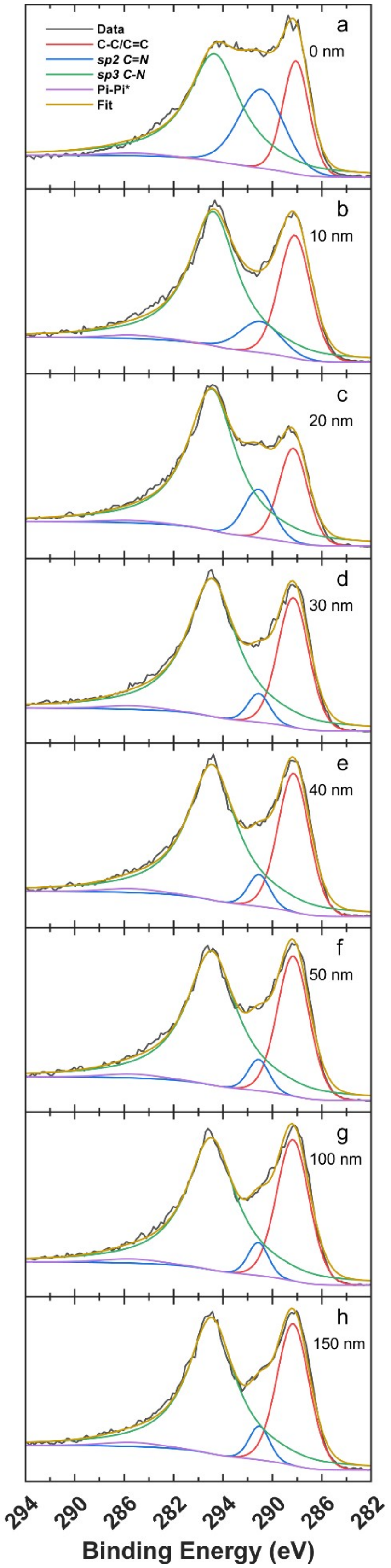

Fig. S2.2.5. For sample DG-9: deconvoluted high resolution C1s scans at depths of a) surface; b) 10 nm; c) 20 nm; d) 30 nm; e) 40 nm; f) 50 nm; g) 100 nm; h) 150 nm.

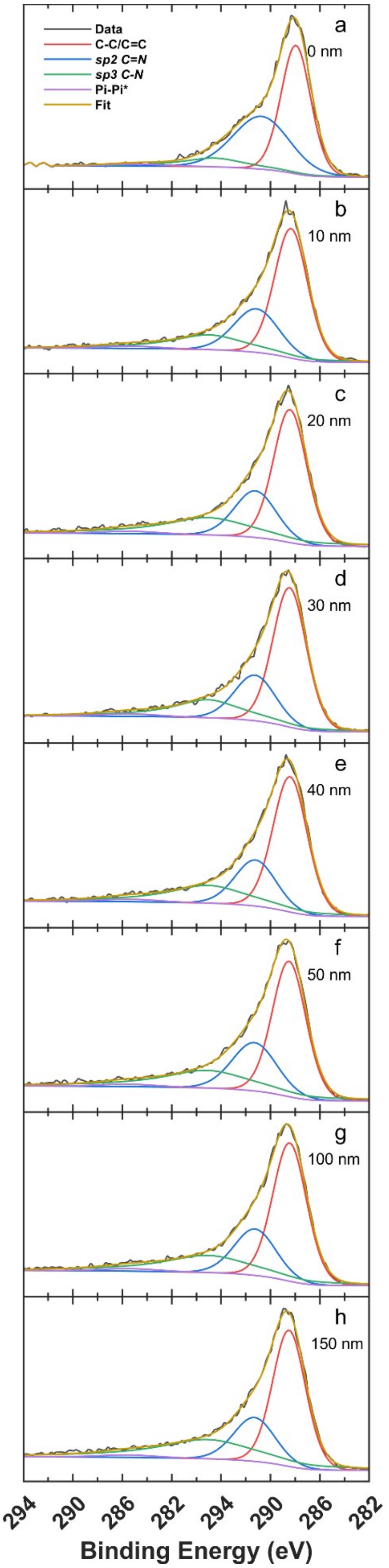

Fig. S2.2.6. For sample DG-12: deconvoluted high resolution C1s scans at depths of a) surface; b) 10 nm; c) 20 nm; d) 30 nm; e) 40 nm; f) 50 nm; g) 100 nm; h) 150 nm.

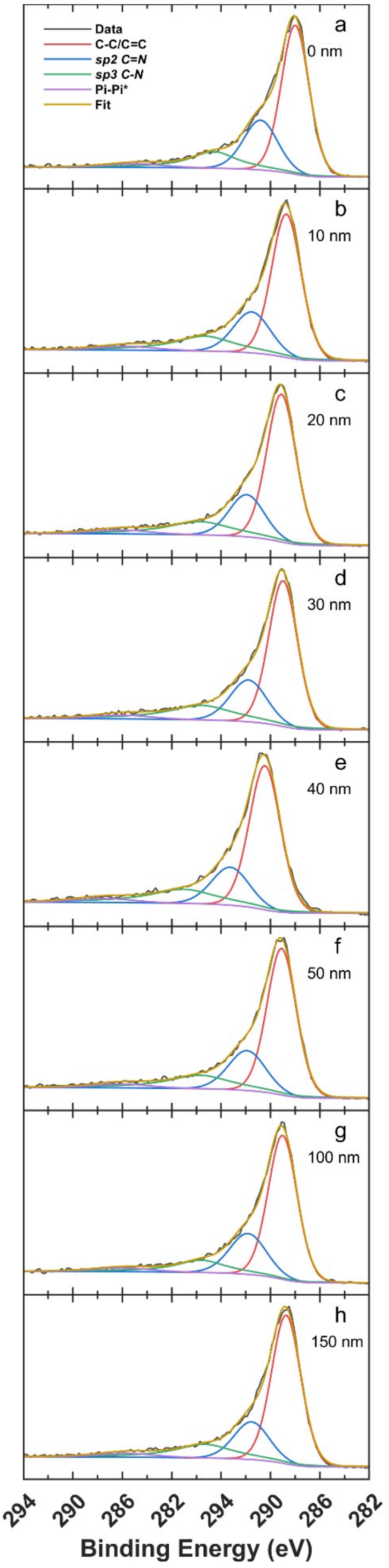

Fig. S2.2.7. For sample DG-99: deconvoluted high resolution C1s scans at depths of a) surface; b) 10 nm; c) 20 nm; d) 30 nm; e) 40 nm; f) 50 nm; g) 100 nm; h) 150 nm.

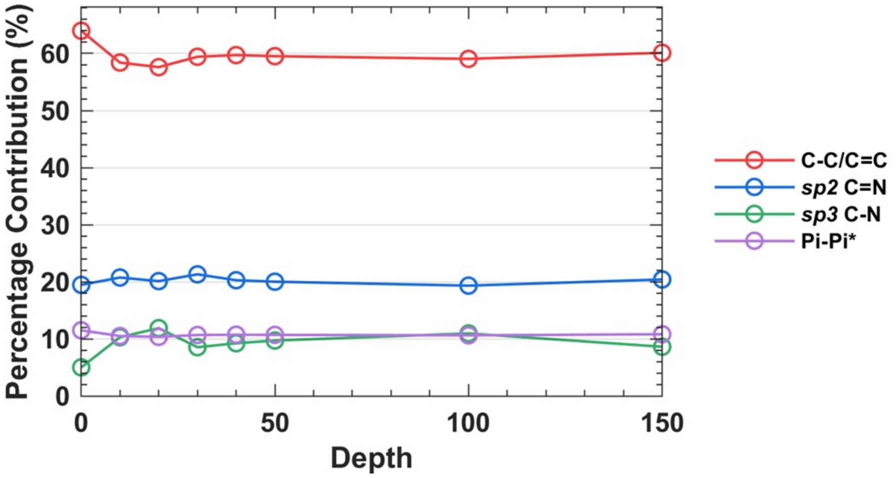

Fig. S2.2.8. For sample DG-0: % contribution of the deconvoluted environments at increasing depth within the sample as measured by XPS.

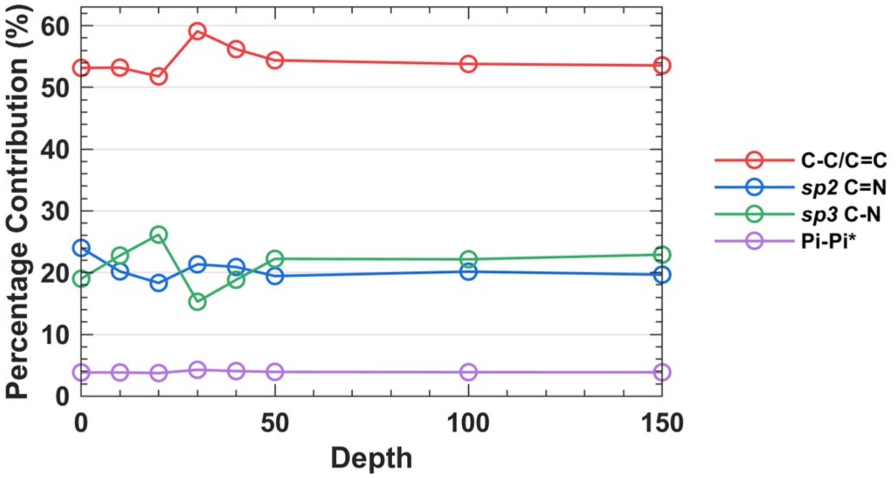

Fig. S2.2.9. For sample DG-1: % contribution of the deconvoluted environments at increasing depth within the sample as measured by XPS.

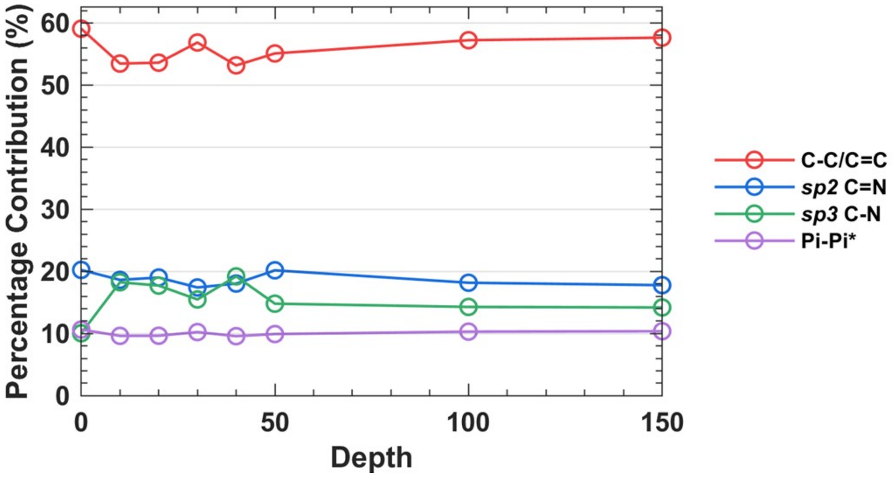

Fig. S2.2.10. For sample DG-3: % contribution of the deconvoluted environments at increasing depth within the sample as measured by XPS.

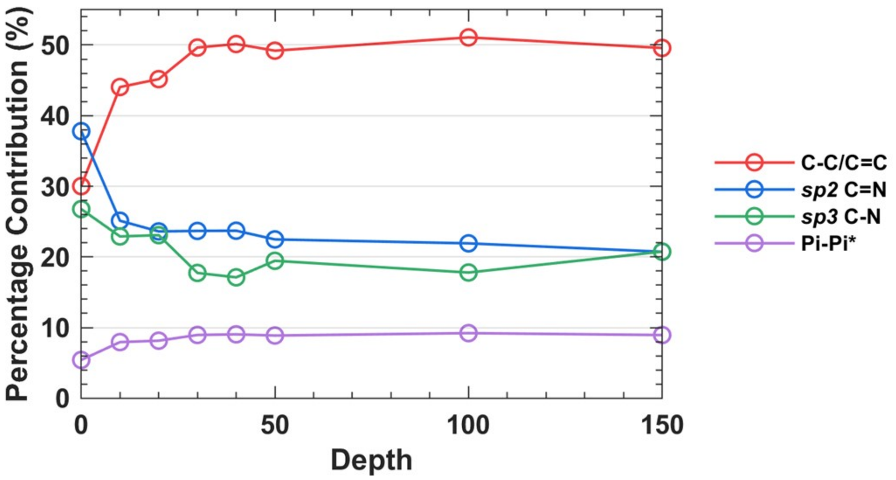

Fig. S2.2.11. For sample DG-6: % contribution of the deconvoluted environments at increasing depth within the sample as measured by XPS.

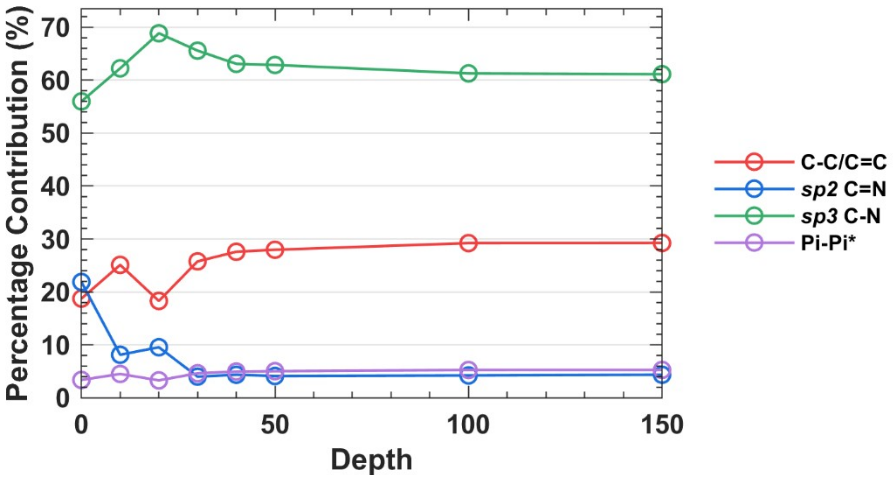

Fig. S2.2.12. For sample DG-9: % contribution of the deconvoluted environments at increasing depth within the sample as measured by XPS.

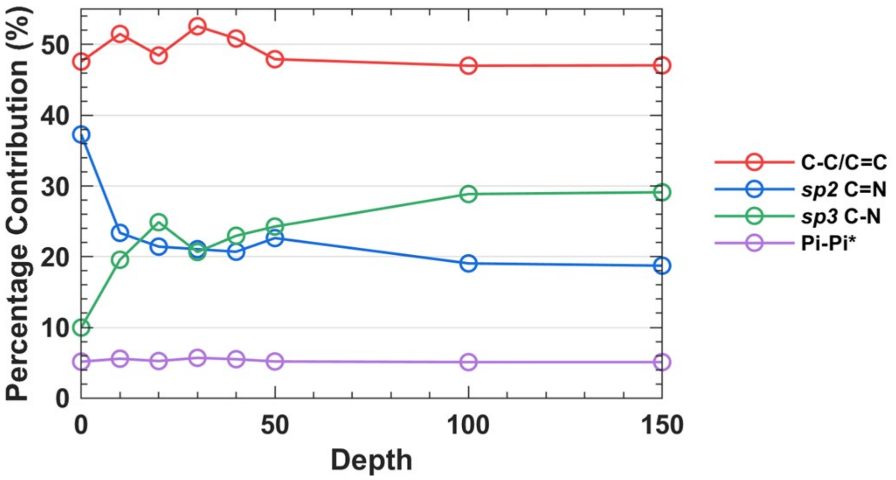

Fig. S2.2.13. For sample DG-12: % contribution of the deconvoluted environments at increasing depth within the sample as measured by XPS.

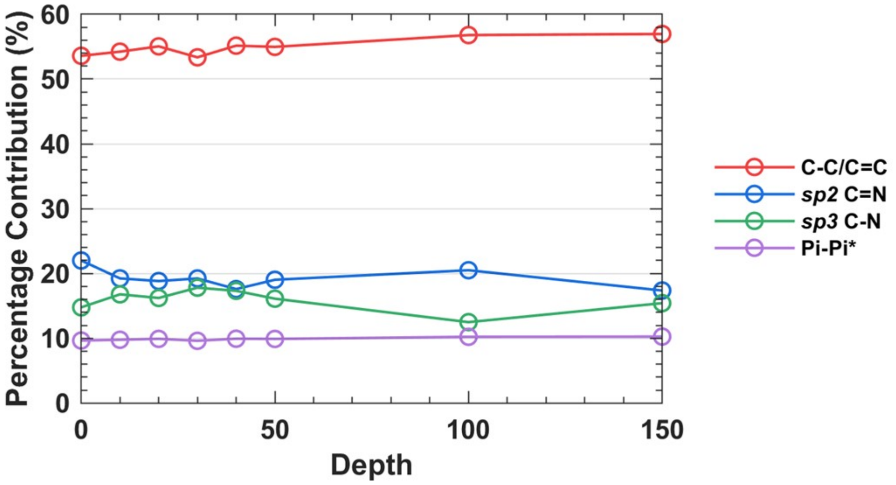

Fig. S2.2.14. For sample DG-99: % contribution of the deconvoluted environments at increasing depth within the sample as measured by XPS.

S2.3 High Resolution N1s Surface Scans

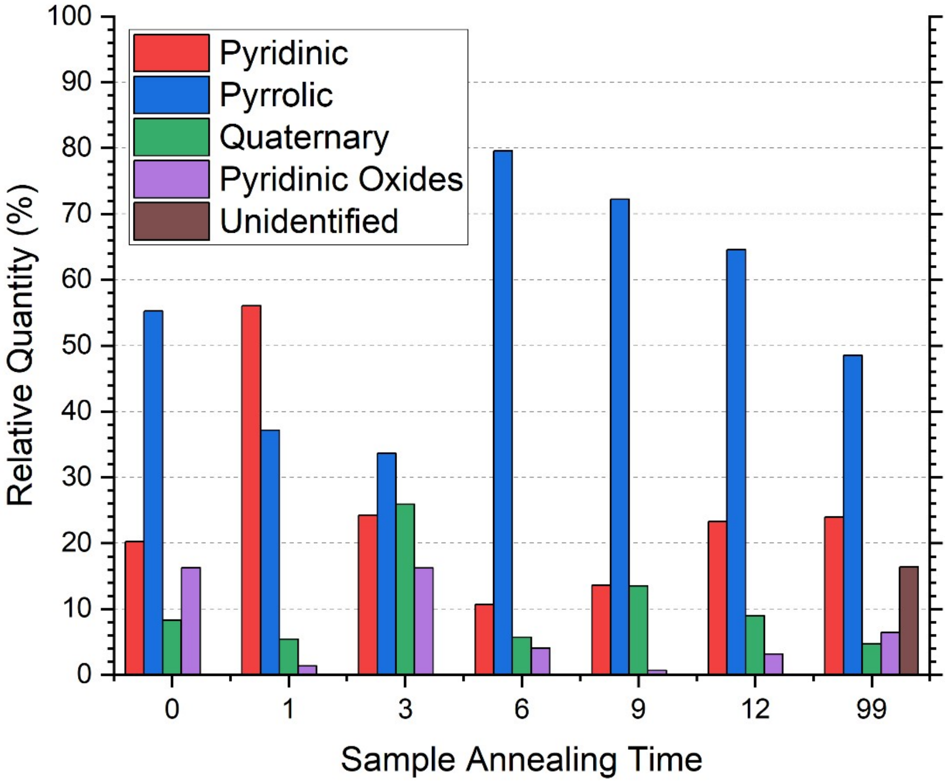

Fig. S2.3.1. Overall deconvoluted N1s surface results for all samples, showing a wide variety of environments across annealing times. Generally pyrrolic environments were dominant, a low concentration of oxides and quaternary (graphitic) environments were generally observed.

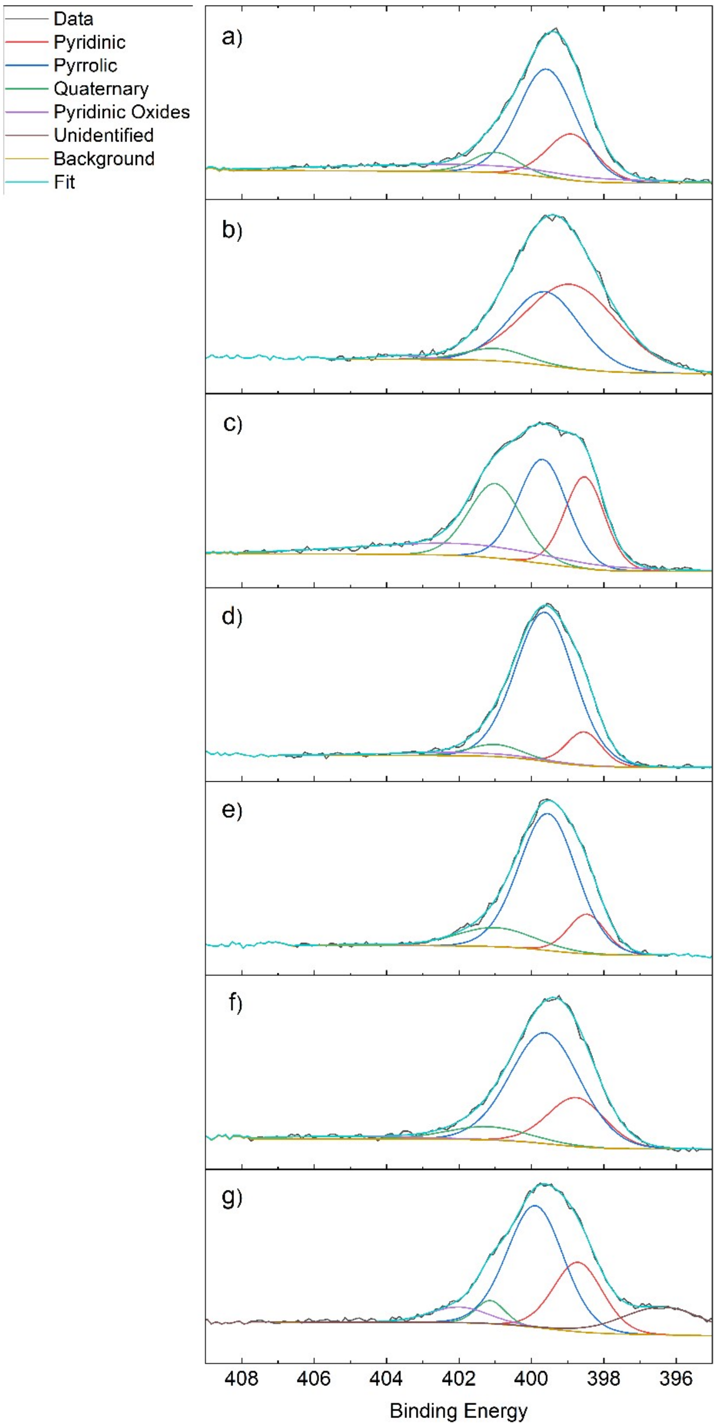

Fig. S2.3.2. Overall deconvoluted N1s surface spectra for all samples, an unidentified peak was observed for DG-99.

S3. Nuclear Magnetic Resonance

S3.1 ¹H NMR

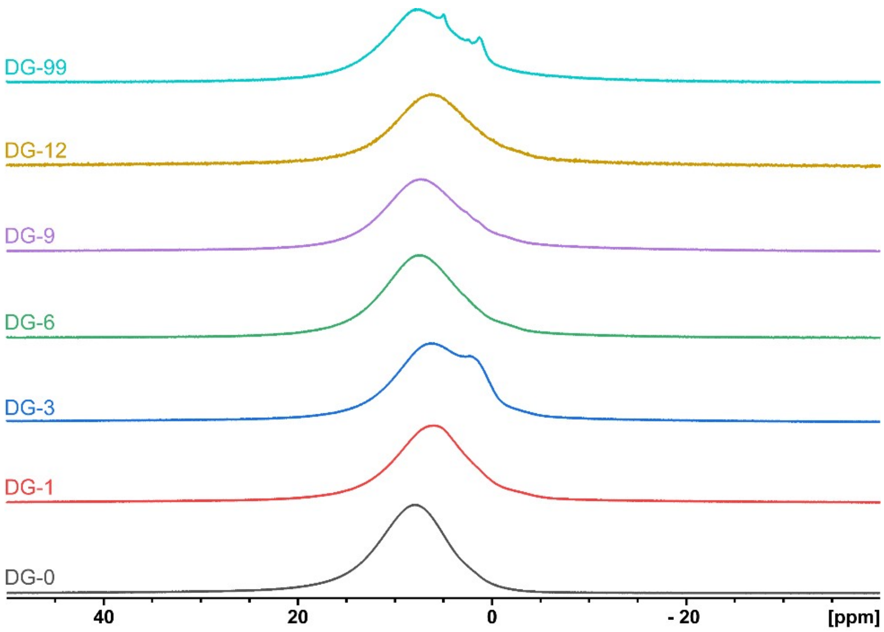

Fig. S3.1.1. ¹H Solid State NMR for all samples.

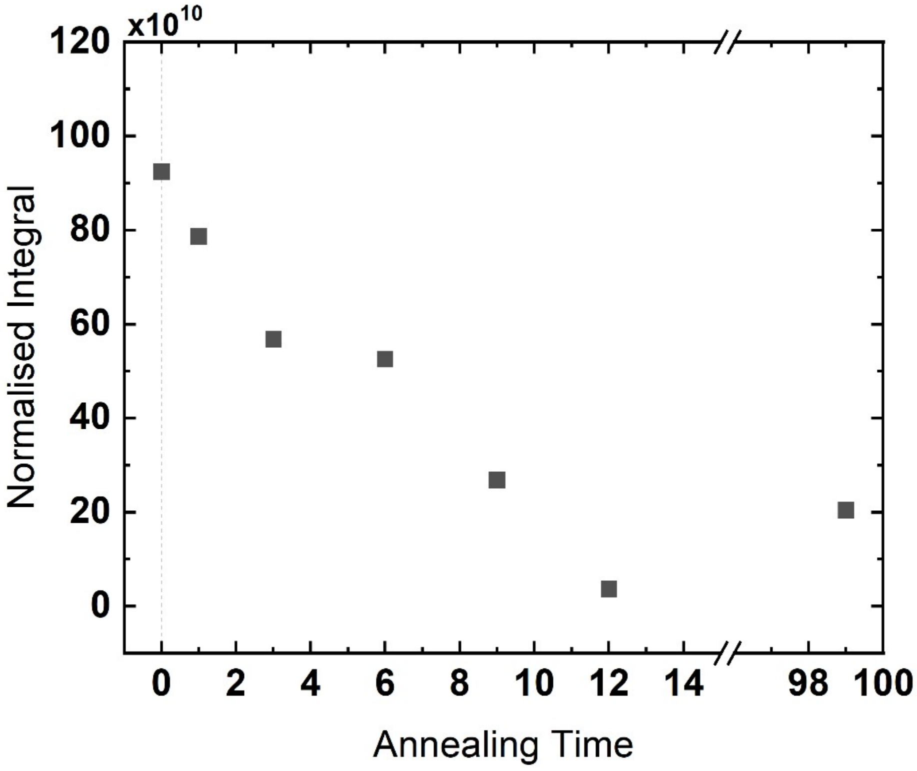

Fig. S3.1.2. Total integrated intensity of peak seen in the ^{1}H SSNMR spectrum, calculated with a Gaussian fit in Topspin using a solid-lineshape analysis.

S3.2 ^{13}C NMR

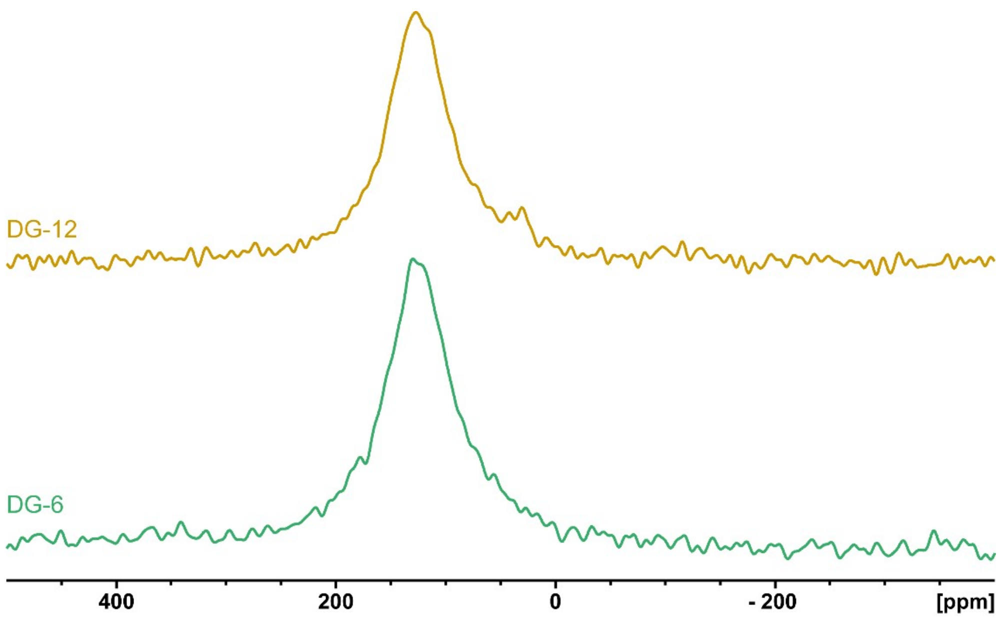

Fig. S3.2.1. ^{13}C Solid State NMR for samples DG-12 and DG-6.

S4. Raman Spectroscopy

Figure S4.1 shows the value of L_a calculated using the relation set out by Ferrari et al:

$$\frac{I(D)}{I(G)} = \frac{C(\lambda)}{L_a}$$

Using a C (514 nm) value of 0.0055 from reference.[1]

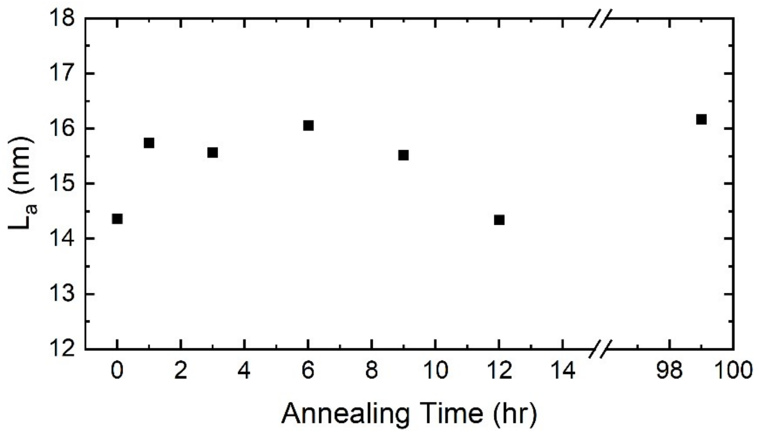

Fig. S4.1. Raman spectroscopy analysis using the relation set out by Ferrari et al. to calculate the in-plane crystallite size, L_a.

In Figure S4.2b and d it can be observed that sample DG-12 has a significantly smaller FWHM(D) and I(D)/I(G) than the other samples. This indicates that a process that results in increased ordering may be occurring at this stage, which is reversed upon extended annealing. At present, this is tentatively attributed to the post-surface saturation loss of nitrogen and ordering occurring in the bulk of the material.

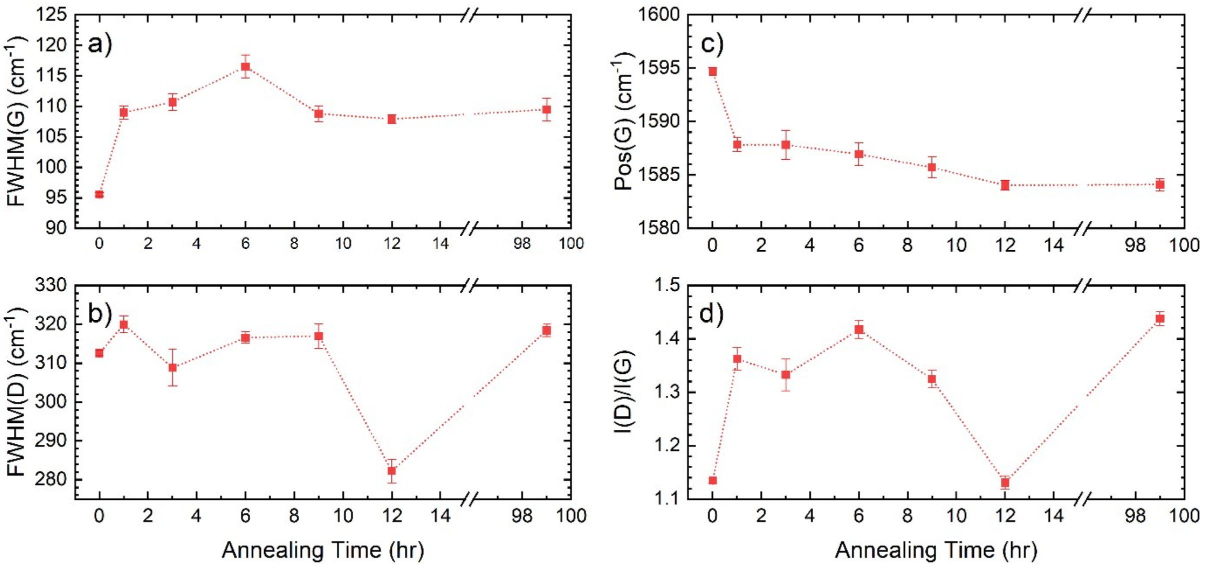

Fig. S4.2. Raman spectroscopy analysis for NDG samples showing the trend across annealing time of a) FWHM(G); b) FWHM(D); c) Pos(G); and d) I(D)/I(G).

S5. Further Electrochemical Testing

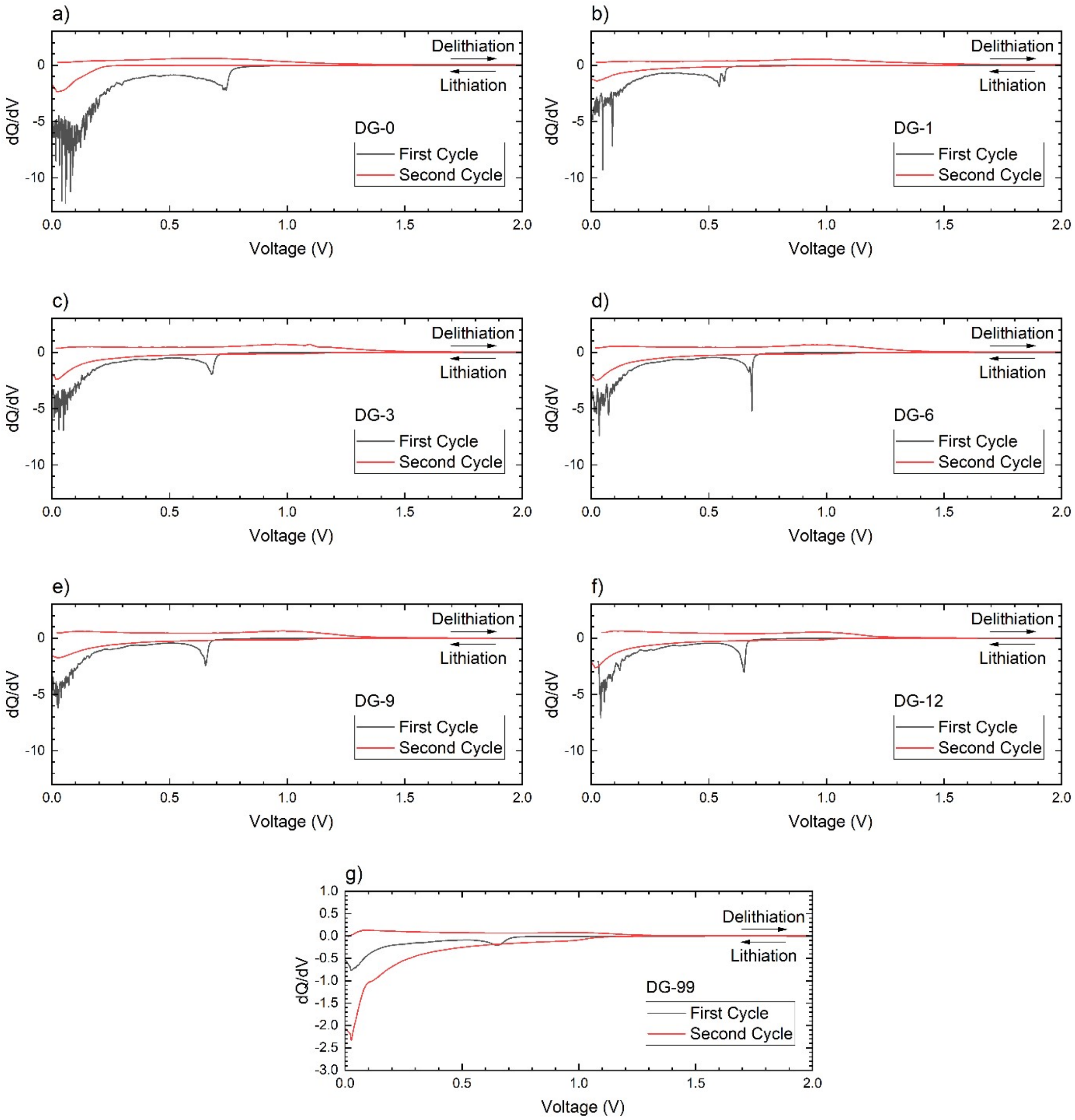

Fig. S5.1. dQ/dV plots for all samples in Li-half cells for the initial discharge and subsequent cycle. a) DG-0; b) DG-1; c) DG-3; d) DG-6; e) DG-9; f) DG-12; g) DG-99. The formation of an SEI can be observed in the first cycle at ca. 0.7 V. Further lithiation generally occurs at low voltages.

A change was made to the way the cells were fabricated, to investigate whether the use of PVDF as a binder affected how the material performed. Carboxymethylcellulose (CMC) was used as a replacement, both PVDF and Super P carbon was not used. Little to no improvement was seen in the capacity at the same cycling rates. Interestingly, however, the cells with material DG-3 performed significantly worse than DG-1, which performed similarly to the cells utilising DG-6. (Fig. 11.) The voltage curves are similar to that of cells fabricated using PVDF and Super P carbon, indicating similar lithiation mechanisms.

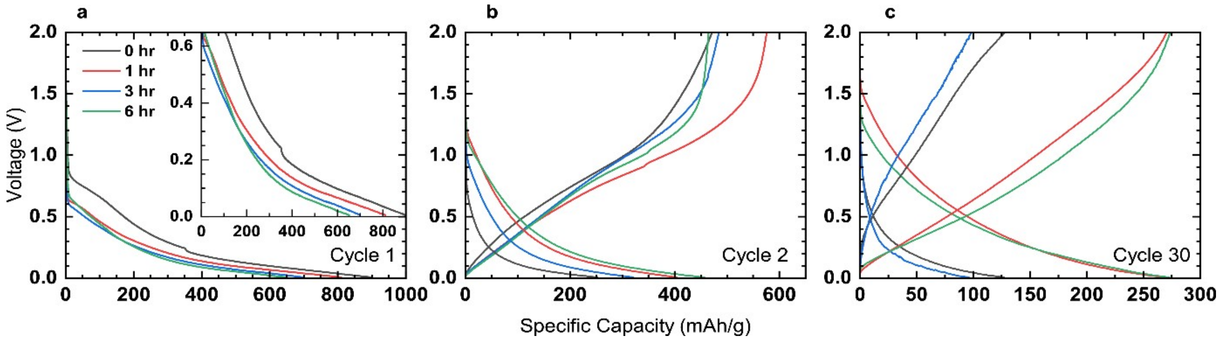

Fig. S5.2. Cycling performance for cells ran with CMC as a replacement binder instead of PVDF. Super P carbon was not included in these cells. a) Cycle 1; b) Cycle 2; c) Cycle 30.

Using a binder of CMC, these materials were tested at high charger rates, as spherical morphology has been suggested to improve storage kinetics. In Figure 12 we see that the capacity retention, after fast charging the cells at incrementally increasing cycling rates, was excellent, as high as 99% for the 6-hour annealed sample. Interestingly, the sample with material annealed for 0 hours showed an increase in capacity across continuous charging rates.

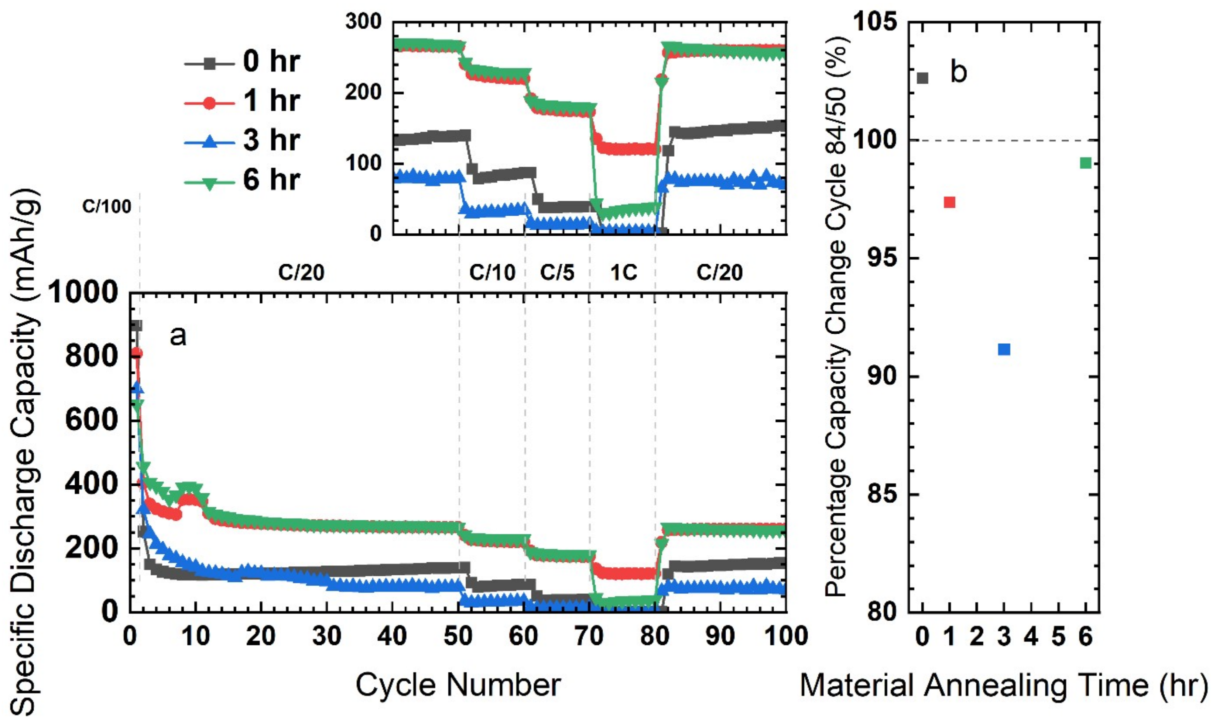

Fig. S5.3. Cycling performance of cells in Li half cells with fast charging (a) over 100 cycles, (b) the % capacity change between cycles 50 and 84. The inset above shows the faster charging region.

S6. Technique for Opening Ampoules

The fabrication of NDG described here involves fabrication of material inside pressurised, fragile quartz ampoules. These ampoules can detonate during the heating process or during the extraction process. A high level of caution should be taken not to over-fill the ampoules, and when obtaining the material. Figure S5.1 describes our methodology, in which the ampoule is opened inside of a fume cupboard from a safe distance. A face shield and leather gloves should be worn.

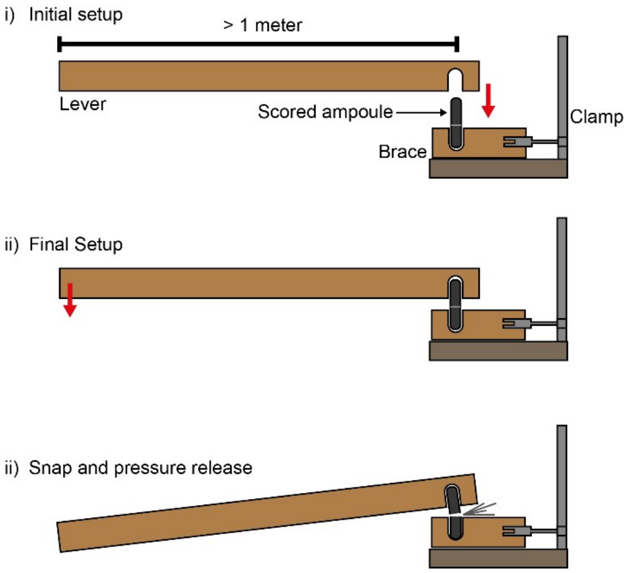

Fig. S6.1. Technique for opening ampoules safely from a distance using a lever system. All stages performed inside a fume cupboard.

1 A. C. Ferrari, *Solid State Commun.*, 2007, **143**, 47–57.
